# Biological Properties of New Chiral 2-Methyl-5,6,7,8-tetrahydroquinolin-8-amine-based Compounds

**DOI:** 10.3390/molecules25235561

**Published:** 2020-11-27

**Authors:** Giorgio Facchetti, Michael S. Christodoulou, Lina Barragán Mendoza, Federico Cusinato, Lisa Dalla Via, Isabella Rimoldi

**Affiliations:** 1 DISFARM, Sezione di Chimica Generale e Organica “A. Marchesini”, Università degli Studi di Milano via Venezian, 21, 20133 Milano, Italy; michail.christodoulou@unimi.it (M.S.C.); isabella.rimoldi@unimi.it (I.R.); 2 Dipartimento di Scienze del Farmaco, Università degli Studi di Padova, via F. Marzolo, 5, 35131 Padova, Italy; lina_barragan@ucol.mx (L.B.M.); federico.cusinato@unipd.it (F.C.); 3 Facultad de Ciencias Químicas, Universidad de Colima, Carr. Colima-Coquimatlán km 9, Coquimatlán 28400, Colima, Mexico

**Keywords:** Schiff bases, ROS production, mitochondrial damage, tetrahydroquinolines, antiproliferative activity

## Abstract

The synthesis of a small library of 8-substituted 2-methyl-5,6,7,8-tetrahydroquinoline derivatives is presented. All the compounds were tested for their antiproliferative activity in non-cancer human dermal microvascular endothelial cells (HMEC-1) and cancer cells: human T-lymphocyte cells (CEM), human cervix carcinoma cells (HeLa), human dermal microvascular endothelial cells (HMEC-1), colorectal adenocarcinoma (HT-29), ovarian carcinoma (A2780), and biphasic mesothelioma (MSTO-211H). Compounds **3a**, **5a**, and **2b**, showing significant IC_50_ values against the whole panel of the selected cells, were further synthesized and tested as pure enantiomers in order to shed light on how their stereochemistry might impact on the related biological effect. The most active compound **(*R*)-5a** was able to affect cell cycle phases and to induce mitochondrial membrane depolarization and cellular ROS production in A2780 cells.

## 1. Introduction

Chiral molecules provide strategic building blocks in the synthesis of pharmaceutical, industrial, and agricultural compounds. It is well known that optically pure chiral compounds stand for being the most abundant among active pharmaceutical drugs whose activity is ascribable to a specific recognition process based on the formation of multiple hydrogen bonds and CH-π interactions with the chiral receptor, thus establishing a diasteromeric relationship with only one enantiomer of the chiral guest [1,2]. Especially in interaction with cellular targets [3], the chirality plays an important role for the biological activities, so, when a chiral center is present in a drug, both the enantiomers must be studied for the evaluation of their pharmacological properties as established by FDA [4,5].

Substituted tetrahydroquinolines are an important moiety present in a wide variety of natural alkaloids [6] and synthetic analogues displaying high biological activities [7,8,9] as potent agents with antimicrobial activities [10,11,12,13,14]. Some of them are also used as ligands in coordinative organometallic complexes, playing a pivotal role in inducing activity at the metal center both for biological and catalytic applications [15,16,17,18,19,20,21,22].

**Scheme 1:**
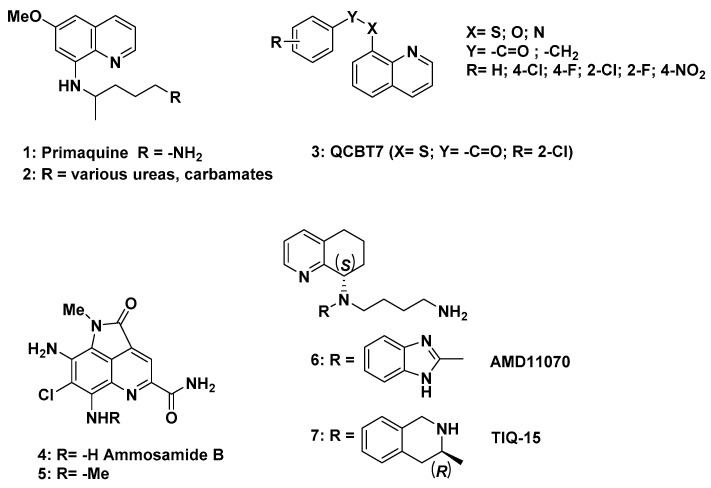
Some examples of anticancer compounds based on the tetrahydroquinoline scaffold.

Recently, some researchers focused their attention on the treatment of tumors with tetrahydroquinoline derivatives and with an 8-amino-quinoline scaffold as potential antiproliferative agents [23,24,25]. The antimalarial drug primaquine (**1**) has shown a pro-oxidant effect on blood, whereas its metabolites have been shown to undergo redox-cycling both in vivo and in vitro [26]. Moreover, several primaquine analogues (**2**) were synthesized and tested for their cytostatic, antiviral, and antioxidant activity (Scheme 1) [27,28]. Proteasome inhibitors, by targeting the cellular machinery involved in protein degradation and homeostasis, stand for an attractive class of anticancer agents showing good efficacy in multiple cancers and currently undergoing clinical trials for the treatment of solid tumors. Quinolin-chlorobenzothiazoate **3** (QCBT7) proved the most cytotoxic of the series with an IC_50_ value of 0.6 µM in HCT-116, showing the ability to address the regulatory subunit of the proteasome instead of the catalytic one and thus being more stable than its precursor quinolin-8-thiol [29]. The pyrroloquinoline scaffold in ammosamide derivatives is indeed fundamental for the chemopreventive potential of such compounds in the treatment of cancer. Ammosamides A and B are naturally occurring metabolites isolated from the marine *Streptomyces* CNR-698 and recognized for their quinone reductase 2 (QR2) inhibitory activity, thus providing cells with protection from chemical damages. Methylation of the 8-amino group in ammosamide B (**4**) affords a compound with increased QR2 inhibitory activity from an IC_50_ of 61 nM (**4**) to an IC_50_ of 4.1 nM (**5**) [30,31]. The identification of compound AMD11070 (**6**) as a potent CXCR4 antagonist shed light on (*S*)-5,6,7,8-tetrahydroquinolin-8-amine as a privileged scaffold for the development of novel compounds not only as anti-HIV drugs but also agents able to inhibit cancer progression via several mechanisms, as CXCR4 is a chemokine receptor expressed on the surface of many cancer cell types. By exchanging the benzimidazole with other heterocycles, it was expected to reduce the off-target effects limiting the use of the compound AMD11070. These efforts led to the discovery of TIQ-15 (**7**), a potent and selective CXCR4 small-molecule inhibitor (IC_50_ of 6.25 nM), which despite its impressive biological profile still suffers from pharmacokinetics instability [32,33]. 

A promising strategy in modern chemotherapy relies on selectively targeting cancer cells depending on the elevated oxidative stress to which they are exposed to as a consequence of their aberrant metabolic activities. Indeed, the increase in the antioxidant ability that cancer cells acquire as an adaptive mechanism for survival makes them extremely responsive to enhanced reactive oxygen species (ROS) levels, potentially allowing them to circumvent multidrug resistance. Amino-quinoline derivatives have recently been reported in the literature for their antiproliferative activity due to their ability to induce mitochondrial dysfunction by increasing ROS levels in HeLaS3 and in the resistant KB-vin cell lines. Both the quinoline core and the presence of amine groups proved essential for their selective and potent anticancer effect [34,35,36]. Based on these supportive findings, we decided to investigate a new series of heterocyclic Schiff bases and their corresponding reduced products starting from 2-methyl-5,6,7,8-tetrahydroquinolin-8-amine [37]. The incorporation of the chiral tetrahydroquinoline moiety could represent an important synthetic strategy in drug discovery for the development of novel chemotherapeutic agents. Starting from these assumptions, a preliminary study was carried out using a racemic form of the synthesized compounds as anticancer agents on the proliferation of human T-lymphocyte cells (CEM), human cervix carcinoma cells (HeLa), and human dermal microvascular endothelial cells (HMEC-1). With the aim to evaluate the potential striking effect subsequent to a different interaction of each enantiomer with biological targets, the most active compounds of the series were synthesized in the enantiopure form and both enantiomers were evaluated for their in vitro antiproliferative activity on three human tumor cell lines (HT-29, A2780, and MSTO-211H). Moreover, for the most active product, **(*R*)-5a**, the mechanism of action responsible for the cytotoxic effect was investigated. In particular, cytofluorimetric analyses were performed to study the effect on the cell cycle and mitochondrial transmembrane potential. The ability to induce intracellular oxidative stress was also evaluated.

## 2. Results and Discussion

**Scheme 2:**
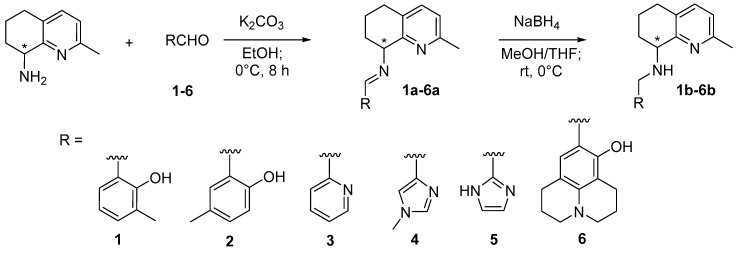
Synthesis of Schiff bases and corresponding amines.

The synthesis of Schiff bases and the corresponding amines proceeded in racemic form as previously reported in the literature starting from salicylaldehyde [37] (Scheme 2). In order to avoid by-product formation as a consequence of the high reactivity of the primary amine of the 2-methyl-5,6,7,8-tetrahydroquinolin-8-amine scaffold, the preparation of the Schiff bases was carried out at 0 °C for 8 h, affording the desired products in good yields (65–70%), without further purification steps. The reduction of the imine group occurred at 0 °C as well but with lower yields (35–40%). The products were then purified by washing extensively the crude residue with *tert*-butyl-methyl ether. 

All the products were fully characterized by ^1^H- and ^13^C-NMR, ESI-MS, and IR. The determination of log *P_ow_* was evaluated for all the compounds by RP-HPLC, equipped with C18 ODS at 25 °C with a water/acetonitrile ratio of 80:20 as eluent (flow 1 mL/min, 254 nm), and using KI as internal standard and commercially available drugs as references [38]. The values of log *P_ow_* resulted in a range of 3.72–4.35, thus proving in accordance with the Lipinski’s rule [39]. The corresponding log D_7.4_ values were calculated using online predictor software, resulting in a range of 1.32–5.59 [40] (https://disco.chemaxon.com/calculators/demo/plugins/logd/).

The obtained racemic compounds were evaluated in an in vitro preliminary screening for their inhibitory effects on the proliferation of three different human cancer cell lines, representative of hematological (CEM, human T-lymphocyte cells) and solid (HeLa, human cervix carcinoma cells) tumors, and on human dermal microvascular endothelial cells (HMEC-1). Combretastatin A4P (CA-4P) is reported as the reference compound [41] (Table 1).

Starting from these preliminary results, the compounds **1a**–**6a** and **1b**–**6b** were further assayed on three human tumor cell lines, specifically HT-29 (colorectal adenocarcinoma), A2780 (ovarian carcinoma), and MSTO-211H (biphasic mesothelioma). Meta-amsacrine (*m*-AMSA) is reported as reference compound [42]and the obtained results are shown in Table 2.

Comparing the results reported in both Table 1 and Table 2, compounds (**3a**, **5a**, and **2b**) proved to exert a significant cytotoxic effect against all the cancer cell lines, and for this reason, they were selected for further evaluation. Indeed, the enantioselective synthesis of the selected compounds was applied in order to evaluate the role of the stereochemistry in the cytotoxicity displayed by the two enantiomers.

The chiral synthesis of the two-ring-fused basic scaffold, the 2-methyl-5,6,7,8-tetrahydroquinolin-8-amine, proceeded as previously reported in the literature for the analogue 8-amino-5,6,7,8-tetrahydroquinoline starting from the DKR by *Candida antarctica* lipase of the 8-hydroxy precursor (for chiral purity, see Supplementary Materials Figure S1) [15,20,43].

The results obtained from the antiproliferative assays are shown in Table 3.

All enantiomers are able to exert a detectable antiproliferative activity on A2780 cells, with IC_50_ values ranging from 5.4 to 17.2 μM. Interestingly, the most effective and the less active are (***R***)-**5a** and (***S***)-**5a**, respectively, and this result highlights a different behavior linked to the enantiomeric form. Conversely, the two chiral forms of **3a** and **2b** do not show any difference in terms of IC_50_, suggesting a similar cytotoxic effect exerted by their respective enantiomers. This behavior was also confirmed on MSTO-211H and indeed, a comparable cytotoxic effect is observed in cells incubated with **(*R*)-3a** and **(*S*)-3a**, while both **(*S*)-2b** and (***R*)-2b** are inactive on this cell line. Otherwise, the enantiomer **(*R*)-5a**, differently from **(*S*)-5a**, which appears ineffective, induces an appreciable inhibition on cell growth.

As regards colorectal adenocarcinoma cells (HT-29), they appeared resistant toward all synthesized compounds, showing IC_50_ values higher than 20 μM under all experimental conditions.

As expected, the IC_50_ values obtained for the reference drug *m*-AMSA are in the submicromolar range for all tested cell lines.

**Figure 1:**
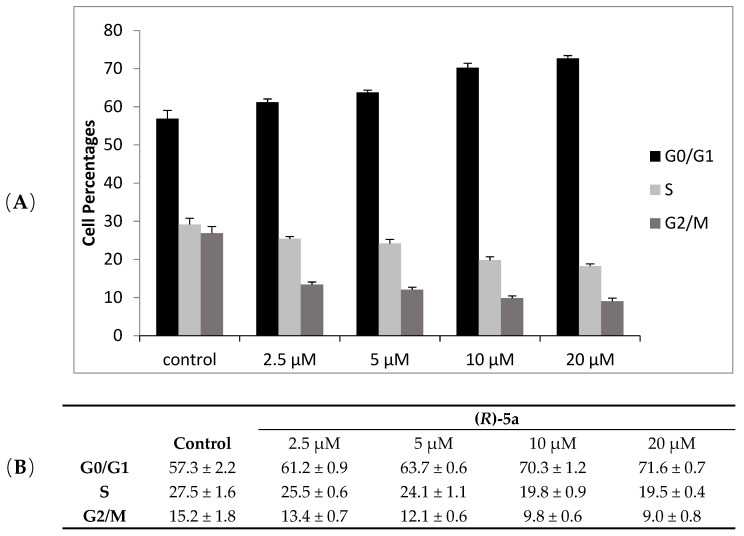
Cell cycle distribution of A2780 cells in the presence of different concentrations of **(*R*)-5a**. Histograms (**A**) and cell percentage values (**B**). Mean values ± SD of four experiments are reported.

The above results prompted us to investigate the mechanism of action of the most interesting **(*R*)-5a.** Based on the crucial role played by DNA and its processing enzymes as targets for many important antiproliferative drugs, we preliminarily investigated the ability of (***R*)-5a** to interact with supercoiled plasmid DNA and to interfere with the catalytic activity of both topoisomerase I and II. Indeed, these two nuclear enzymes are critical for cell growth, being involved in DNA replication, transcription, and chromosome segregation [44]. The obtained results clearly indicated that **(*R*)-5a** is unable to induce any modification in the electrophoretic migration of supercoiled plasmid DNA (see Supplementary Materials Figure S2) as well as any effect on the relaxation ability of both nuclear enzymes (see Supplementary Materials Figure S3), thus excluding such macromolecules as possible targets responsible for its antiproliferative effect. Then, to further investigate the intracellular mechanism of action, the effect on the cell cycle was also analyzed by cytofluorimetric measurements. The most sensitive A2780 cells were incubated in the presence of increasing concentrations of test compound and loaded with propidium iodide. The obtained results are shown in Figure 1.

The data reported in Figure 1 indicate that the treatment of cells with **(*R*)-5a** induces some changes in the cell cycle phases. In detail, a concentration-dependent increase can be observed in G0/G1 phase with respect to the control condition, i.e., untreated cells, accompanied by a concurrent decrease of both S and G2/M. Based on the crucial role proposed for mitochondria in cell cycle progression and in particular for G1 to S transition [45], we further investigated the mechanism of action by studying the effect of **(*R*)-5a** on the mitochondrial transmembrane potential in cells treated with test compound.

**Figure 2:**
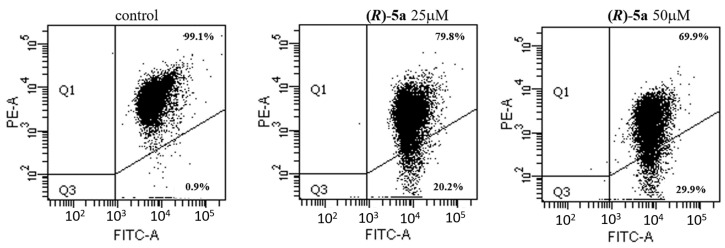
Mitochondrial transmembrane potential in A2780 cells treated with **(*R*)-5a** at indicated concentrations. A representative experiment is shown.

For this purpose, A2780 cells were incubated in the presence of different concentrations of **(*R*)-5a** and then treated with JC-1, a fluorescent cationic probe able to enter into mitochondrial matrix, as a function of mitochondrial transmembrane potential [46]. The depolarization of the organelle membrane, as result of a mitochondrial-damaging agent, can be detected by a decrease in fluorescence emission. Figure 2 reports the effect of **(*R*)-5a** on the mitochondrial membrane potential in A2780 cells incubated with 25 and 50 μM of the test compound.

The incubation of A2780 cells with **(*R*)-5a** provokes a mitochondrial depolarization that depends on the concentration. In detail, about 20% of cells showed a decrease in fluorescence emission at the 25 μM concentration and such a percentage increases up to about 30% at the higher concentration taken into consideration (50 μM), thus indicating for **(*R*)-5a** the ability to affect mitochondrial functionality.

**Figure 3:**
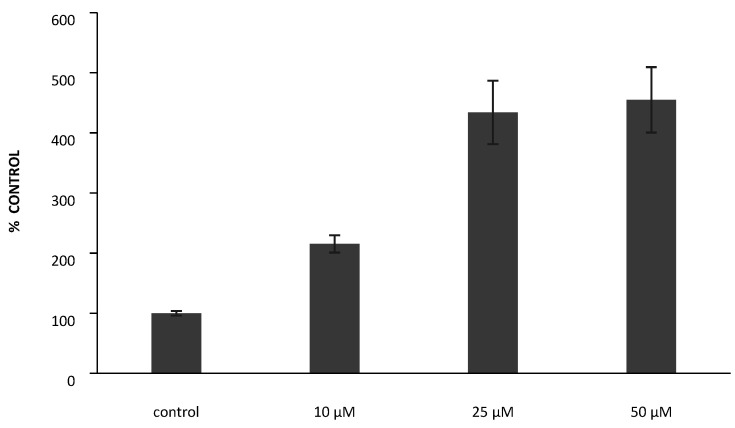
ROS production in A2780 cells after 120 min of treatment with **(*R*)-5a** at indicated concentrations. (*n* = 3–5, mean ± standard deviation).

It is well known that mitochondria are the main intracellular source of ROS. Because ROS, by promoting oxidative damage in susceptible targets, such as crucial proteins and the phospholipid cardiolipin [47], can lead to a loss of mitochondrial membrane potential and apoptosis [48], we evaluated the ability of **(*R*)-5a** to induce short-term (0–120 min) ROS production in A2780 cells stained with the cell-permeable fluorogenic probe dichlorodihydrofluorescein diacetate (Figure 3).

The results shown in Figure 3 clearly indicate that the test compound increases the basal ROS in ovarian cancer cells. In particular, after 120 min of incubation, a concentration-dependent production of ROS is observed up to the 25 μM concentration, while at 50 μM, a plateau seems to be reached. Interestingly, at this latter concentration, **(*R*)-5a** is able to induce an intracellular amount of ROS more than 4-fold higher with respect to that measured in untreated cells (control). These data suggest for **(*R*)-5a** the ability to promote significant intracellular oxidative stress, which could contribute to the cytotoxic effect exerted by the compound.

## 3. Experimental

All manipulations involving-air sensitive materials were carried out in an inert atmosphere glove box or using standard Schlenk line techniques, under an atmosphere of nitrogen or argon in oven-dried glassware. All tested compounds possessed a purity of >98% confirmed via elemental analyses (CHN) with a Perkin Elmer 2400 instrument. ^1^H-NMR and ^13^C-NMR spectra were recorded on a Bruker DRX Avance 300 MHz equipped with a non-reverse probe (BBI) and also on a Bruker DRX Avance 600 MHz. MS analyses were performed by using a Thermo Finnigan (San Jose, CA, USA) LCQ Advantage system MS spectrometer (San Jose, CA, USA) with an electrospray ionization source and an ‘Ion Trap’ mass analyser. The MS spectra were obtained by direct infusion of a sample solution in MeOH under ionization, ESI positive. Log *P_ow_*’s values and purity were evaluated with Partisil C18-ODS reversed-phase HPLC column with Merck-Hitachi L-7100 equipped with Detector UV6000LP. (***R***)- and (***S***)-2-methyl-5,6,7,8-tetrahydroquinolin-8-amine were synthesized according to the procedure reported in the literature [15,20,43,49].

### 3.1. General Procedure for the Synthesis of Compounds ***1a**–**6a***

2-Methyl-5,6,7,8-tetrahydroquinolin-8-amine (1.2 equivalent) was dissolved in EtOH (10 mL) and aldehyde (1 equivalent) was added at 0 °C. The reaction was stirred for 8 h at 0 °C and then water (5 mL) was added. The aqueous solution was extracted with CH_2_Cl_2_ (3 × 10 mL). The combined organic layers were dried over anhydrous Na_2_SO_4_ and the solvent was removed under vacuum. The obtained product did not need further purification.

(*E*)-2-methyl-6-(((2-methyl-5,6,7,8-tetrahydroquinolin-8-yl)imino)methyl)phenol **1a**: white solid. Yield 70%. m.p. 102.2–103.8 °C. ^1^H NMR (CDCl_3_, 300 MHz) δ: 8.62 (s, 1H, H_8_), 7.31 (d, *J* = 7.6 Hz, 1H, H_3_), 7.16 (m, 2H, H_2_ + H_11_), 6.95 (d, *J* = 7.3 Hz, 1H, H_9_), 6.78 (t, *J* = 6.8 Hz, 1H, H_10_), 4.56 (m, 1H, H_7_), 2.82 (m, 2H, H_4_), 2.49 (s, 3H, H_1_), 2.17 (s, 3H, H_12_), 2.16 (m, 2H, H_6_), 1.90 (m, 2H, H_5_) ppm. ^13^C NMR (CDCl_3_, 75 MHz) δ: 163.94, 159.52, 155.93, 153.81, 137.79, 133.04, 129.18, 129.07, 125.75, 122.97, 118.33, 118.14, 68.89, 31.13, 28.14, 24.12, 18.49, 16.66 ppm; FTIR (NaCl) v = 3421.65, 2934.74, 1622.85, 1197.97, 747.89 cm^−1^; MS (ESI) for C_18_H_20_N_2_O *m/z* 281.31 [M + H]^+^. Anal. Calcd for C_18_H_20_N_2_O: C, 77.11; H, 7.11; N, 9.99. Found: C, 77.33; H, 7.26, N, 9.91.

(*E*)-4-methyl-2-(((2-methyl-5,6,7,8-tetrahydroquinolin-8-yl)imino)methyl)phenol **2a**: white solid. Yield 67%. m.p. 101.8–103.4 °C. ^1^H NMR (CDCl_3_, 300 MHz) δ: 8.55 (s, 1H, H_8_), 7.30 (d, *J* = 7.5 Hz, 1H, H_3_), 7.09 (m, 2H, H_2_ + H_10_), 6.96 (d, *J* = 7.0 Hz, 1H, H_9_), 6.81 (d, *J* = 6.7 Hz, 1H, H_11_), 4.55 (t, *J* = 3 Hz, 1H, H_7_), 2.81 (m, 2H, H_4_ ), 2.49 (s, 3H, H_1_), 2.29 (s, 3H, H_12_), 2.10 (m, 2H, H_6_), 1.88 (m, 2H, H_5_) ppm. ^13^C NMR (CDCl_3_, 75 MHz) δ: 164.63, 158.85, 155.89, 153.79, 137.41, 132.79, 131.41, 129.24, 127.44, 122.35, 118.78, 116.56, 66.87, 31.06, 28.11, 23.65, 20.85, 18.42 ppm; FTIR (NaCl) v = 3420.85, 2934.12, 1618.43, 1199.09, 745.92 cm^−1^; MS (ESI) for C_18_H_20_N_2_O *m/z* 281.29 [M + H]^+^. Anal. Calcd for C_18_H_20_N_2_O: C, 77.11; H, 7.11; N, 9.99. Found: C, 77.34; H, 7.25, N, 9.91.

(*E*)-*N*-(2-methyl-5,6,7,8-tetrahydroquinolin-8-yl)-1-(pyridin-2-yl)methanimine **3a**: orange solid. Yield 65%. m.p. 102.8–104.3 °C. ^1^H NMR (CDCl_3_, 300 MHz) δ: 8.51 (d, *J* = 8.7 Hz, 1H, H_12_), 8.42 (s, 1H, H_8_), 7.89 (d, *J* = 7.9 Hz, 1H, H_10_), 7.56 (t, *J* = 6 Hz, 1H, H_9_), 7.16 (m, 2H, H_3_ + H_11_), 6.83 (d, *J* = 7.0 Hz, 1H, H_2_), 4.61 (t, *J* = 3 Hz, 1H, H_7_), 2.69 (m, 2H, H_4_), 2.39 (s, 3H, H_1_), 2.05 (m, 2H, H_6_), 1.72 (m, 2H, H_5_) ppm. ^13^C NMR (CDCl_3_, 75 MHz) δ: 160.82, 155.80, 154.93, 154.41, 149.41, 138.57, 136.74, 129.54, 125.58, 122.28, 121.55, 68.59, 31.15, 28.34, 23.63, 18.59 ppm; FTIR (NaCl) v = 3391.41, 2924.16, 1945.19, 1735.64, 1591.47, 1197.30, 792.06 cm^−1^; MS (ESI) for C_16_H_17_N_3_ *m/z* 252.34 [M + H]^+^. Anal. Calcd for C_16_H_17_N_3_: C, 76.46; H, 6.82; N, 16.72. Found: C, 76.68; H, 6.90, N, 16.65. *S*-isomer: [α]^20^_D_ = −39.1 (c = 0.5; CHCl_3_). Anal. Calcd for C_16_H_17_N_3_: C, 76.46; H, 6.82; N, 16.72. Found: C, 76.70; H, 6.91, N, 16.63.; *R*-isomer: [α]^20^_D_ = +33.2 (c = 0.3; CHCl_3_). Anal. Calcd for C_16_H_17_N_3_: C, 76.46; H, 6.82; N, 16.72. Found: C, 76.72; H, 6.93, N, 16.61. 

(*E*)-1-(1-methyl-1H-imidazol-4-yl)-*N*-(2-methyl-5,6,7,8-tetrahydroquinolin-8-yl)methanimine **4a**: white solid. Yield 70%. m.p. 103.2–104.5 °C. ^1^H NMR (CDCl_3_, 300 MHz) δ: 8.37 (s, 1H, H_8_), 7.59 (s, 1H, H_10_), 7.52 (s, 1H, H_12_), 7.40 (d, *J* = 7.3 Hz, 1H, H_3_), 6.91 (d, *J* = 6.9 Hz, 1H, H_2_), 4.52 (t, *J* = 6 Hz, 1H, H_7_), 3.67 (s, 3H, H_11_), 2.77 (m, 2H, H_4_), 2.44 (s, 3H, H_1_), 2.11 (m, 2H, H_6_) 1.78 (m, 2H, H_5_) ppm. ^13^C NMR (CDCl_3_, 75 MHz) δ: 156.77, 156.57, 154.94, 142.21, 138.14, 137.37, 130.26, 121.89, 119.13, 69.60, 34.09, 31.35, 28.39, 24.74, 18.72 ppm; FTIR (NaCl) v = 3420.09, 2917.64, 2230.65, 1639.73, 1197.78 cm^−1^; MS (ESI) for C_15_H_18_N_4_ *m/z* 255.20 [M + H]^+^. Anal. Calcd for C_15_H_18_N_4_: C, 70.84; H, 7.13; N, 22.03. Found: C, 71.07; H, 7.20, N, 21.87.

(*E*)-1-(1H-imidazol-2-yl)-*N*-(2-methyl-5,6,7,8-tetrahydroquinolin-8-yl)methanimine **5a**: white solid. Yield 69%. m.p. 101.2–102.6 °C. ^1^H NMR (CDCl_3_, 300 MHz) δ: 8.33 (s, 1H, H_8_), 7.28 (m, 3H, H_3_ + H_11_ + H_12_), 6.96 (d, *J* = 8.5 Hz, 2H, H_12_ + H_10_), 4.60 (m, 1H, H_7_), 2.75 (m, 2H, H_4_), 2.45 (s, 3H, H_1_), 2.06 (m, 2H, H_6_), 1.80 (m, 2H, H_5_) ppm. ^13^C NMR (CDCl_3_, 75 MHz) δ: 156.95, 154.09, 152.55, 144.86, 139.64, 129.53, 127.85, 123.26, 122.29, 69.37, 31.19, 27.90, 23.92, 17.13 ppm; FTIR (NaCl) v = 3419.92, 2905.17, 2222.55, 1631.70, 1188.45 cm^−1^; MS (ESI) for C_14_H_16_N_4_ *m/z* 241.14 [M + H]^+^. Anal. Calcd for C_14_H_16_N_4_: C, 69.97; H, 6.71; N, 23.31. Found: C, 70.23; H, 6.81, N, 23.08. *S*-isomer: [α]^20^_D_ = −36.8 (c = 0.4; CHCl_3_). Anal. Calcd for C_14_H_16_N_4_: C, 69.97; H, 6.71; N, 23.31. Found: C, 70.20; H, 6.81, N, 23.10. *R*-isomer: [α]^20^_D_ = +34.2 (c = 0.5; CHCl_3_). Anal. Calcd for C_14_H_16_N_4_: C, 69.97; H, 6.71; N, 23.31. Found: C, 70.25; H, 6.83, N, 23.07.

(*E*)-9-(((2-methyl-5,6,7,8-tetrahydroquinolin-8-yl)imino)methyl)-2,3,6,7-tetrahydro-1*H*,5*H*-pyrido [3,2,1-ij]quinolin-8-ol **6a**: yellow solid. Yield 65%. m.p. 104.2–105.8 °C. ^1^H NMR (CDCl_3_, 300 MHz) δ: 8.17 (s, 1H, H_8_), 7.28 (d, *J* = 7.6 Hz, 1H, H_3_), 6.93 (d, *J* = 7.0 Hz, 1H, H_2_), 6.64 (s, 1H, H_9_), 4.49 (t, *J* = 3 Hz, 1H, H_7_), 3.19 (dd, *J* = 6 Hz, *J* = 6.3 Hz, 4H, H_10_ + H_11_), 2.77 (m, 2H, H_6_), 2.65 (dd, *J* = 6 Hz, *J* = 6.3 Hz, 5H, H_14_ + H_15_), 2.44 (s, 3H, H_1_), 2.11 (m, 3H, H_4_ + H_5_), 1.95 (m, 5H, H_4_ + H_12_ + H_13_) ppm. ^13^C NMR (CDCl_3_, 75 MHz) δ: 162.58, 161.92, 156.27, 153.73, 146.49, 137.47, 129.11, 128.30, 122.22, 111.51, 108.00, 106.80, 65.62, 50.09, 49.79, 31.02, 28.07, 27.30, 24.13, 22.21, 21.69, 20.34, 18.38 ppm; FTIR (NaCl) v = 3421.15, 2917.25, 2849.22, 1955.69, 1605.73, 1467.52, 1429.23, 1198.08, 860.19, 791.02 cm^−1^; MS (ESI) for C_23_H_27_N_3_O *m/z* 362.41 [M + H]^+^. Anal. Calcd for C_23_H_27_N_3_O: C, 76.42; H, 7.53; N, 11.62. Found: C, 76.65; H, 7.61, N, 11.54.

### 3.2. General Procedure for the Synthesis of Compounds ***1b**–**6b***

The Schiff base (1 equivalent) was dissolved in a mixture of MeOH/THF (1:1.5 mL) and cooled to 0 °C. NaBH_4_ (0.5 equivalent) was added and the mixture was stirred for 1 h at room temperature. The solution was quenched with saturated NH_4_Cl solution (4 mL) and extracted with CH_2_Cl_2_ (3 × 10 mL). Combined organic layers were dried over Na_2_SO_4_ and the solvent was evaporated. The crude was purified by washing it extensively with *tert*-butyl-methyl ether. The ethereal phase was then separated from an insoluble residue by filtration and the solvent removed under vacuum, affording the desired product. 

2-methyl-6-(((2-methyl-5,6,7,8-tetrahydroquinolin-8-yl)amino)methyl)phenol **1b**: yellow solid. Yield 69%. m.p. 89.2–91.1 °C. ^1^H NMR (CDCl_3_, 300 MHz) δ: 7.30 (d, *J* = 7.7 Hz, 1H, H_3_), 7.04 (d, *J* = 7.1 Hz, 1H, H_2_), 6.96 (m, 2H, H_9_+H_11_), 6.73 (t, *J* = 9 Hz, *J* = 5.7 Hz, 1H, H_10_), 4.14 (dd, *J* = 12.5 Hz, 2H, H_8_), 3.82 (t, *J* = 6.5 Hz, 1H, H_7_), 2.75 (m, 2H, H_4_), 2.51 (s, 3H, H_12_), 2.25 (s, 3H, H_3_), 1.91 (m, 2H, H_6_), 1.78 (m, 2H, H_5_) ppm. ^13^C NMR (CDCl_3_, 75 MHz) δ: 156.22, 155.52, 155.10, 137.47, 129.68, 129.13, 125.79, 125.23, 123.22, 121.52, 117.79, 57.94, 50.82, 28.55, 28.28, 24.53, 19.84, 16.27 ppm; FTIR (NaCl) v = 3413.38, 3283.17, 2925.27, 2855.76, 1596.54, 1573.38, 1471.07, 1255.75, 1230.91, 1198.08, 1082.72, 815.13, 764.63, 741.53 cm^−1^; MS (ESI) for C_18_H_22_N_2_O *m/z* 283.23 [M + H]^+^. Anal. Calcd for C_18_H_22_N_2_O: C, 76.56; H, 7.85; N, 9.92. Found: C, 76.79; H, 7.94, N, 9.85.

4-methyl-2-(((2-methyl-5,6,7,8-tetrahydroquinolin-8-yl)amino)methyl)phenol **2b**: pale yellow solid. Yield 68%. m.p. 89.5–90.8 °C. ^1^H NMR (CDCl_3_, 300 MHz) δ: 7.30 (d, *J* = 6.7 Hz, 1H, H_3_), 6.97 (d, *J* = 9 Hz, 2H, H_2_ + H_9_), 6.87 (s, 1H, H_10_), 6.73 (d, *J* = 6.7 Hz ,1H, H_11_), 4.10 (dd, *J* = 12.5 Hz 2H, H_8_), 3.80 (m, 1H, H_7_), 2.74 (m, 2H, H_4_), 2.52 (s, 3H, H_12_), 2,27 (s, 3H, H_1_), 2.15 (m, 2H, H_5_), 1.93 (m, 2H, H_6_) ppm. ^13^C NMR (CDCl_3_, 75 MHz) δ: 155.75, 155.47, 155.15, 137.51, 129.15, 128.92, 128.01, 123.73, 122.44, 117.08, 57.94, 50.79, 28.72, 28.24, 24.06, 20.47, 19.81 ppm; FTIR (NaCl) v = 3281.66, 2925.60, 2858.02, 1869.90, 1597.70, 1499.73, 1471.66, 1258.47, 1091.84, 909.07, 816.23, 732.33 cm^−1^; MS (ESI) for C_18_H_22_N_2_O *m/z* 283.26 [M + H]^+^. Anal. Calcd for C_18_H_22_N_2_O: C, 76.56; H, 7.85; N, 9.92. Found: C, 76.80; H, 7.93, N, 9.84. *S*-isomer: [α]^20^_D_ = -16.8 (c = 0.7; CHCl_3_). Anal. Calcd for C_18_H_22_N_2_O: C, 76.56; H, 7.85; N, 9.92. Found: C, 76.83; H, 7.95, N, 9.87. *R*-isomer: [α]^20^_D_ = +14.3 (c = 0.3; CHCl_3_). Anal. Calcd for C_18_H_22_N_2_O: C, 76.56; H, 7.85; N, 9.92. Found: C, 76.81; H, 7.94, N, 9.82.

2-methyl-*N*-(pyridin-2-ylmethyl)-5,6,7,8-tetrahydroquinolin-8-amine **3b**: white solid. Yield 66%. m.p. 87.8–89.2 °C. ^1^H NMR (CDCl_3_, 300 MHz) δ: 8.57 (d, *J* = 8.5 Hz, 1H, H_12_), 7.65 (ddd, *J* = 6.0 Hz, 3.0 Hz, 1H, H_10_), 7.46 (d, *J* = 7.6 Hz, 1H, H_3_), 7.26 (d, *J* = 7.5 Hz, 1H, H_9_), 7.16 (ddd, *J* = 6.0 Hz, 3.0 Hz, 1H, H_11_), 6.93 (d, *J* = 7.1 Hz, 1H, H_2_), 4.15 (d, *J* = 3.0 Hz, 2H, H_8_), 3.91 (dd, *J* = 8.0 Hz, 3.0 Hz, 1H, H_7_), 2.74 (m, 2H, H_4_), 2.48 (s, 3H, H_1_), 2.22 (m, 1H, H_5_), 2.05 (m, 1H, H_5_), 1.91 (m, 1H, H_6_), 1.73 (m, 2H, H_6_) ppm. ^13^C NMR (CDCl_3_, 75 MHz) δ: 151.80, 150.90, 150.43, 147.14, 136.75, 132.11, 127.22, 123.58, 121.28, 119.25, 58.59, 50.32, 30.05, 26.43, 21.33, 17.15 ppm; FTIR (NaCl) v = 3389.14, 2898.61, 1933.94, 1695.99, 1568.12, 1182.40, 789.96 cm^−1^; MS (ESI) for C_16_H_19_N_3_ *m/z* 254.26 [M + H]+. Anal. Calcd for C_16_H_19_N_3_: C, 75.85; H, 7.56; N, 16.59. Found: C, 76.08; H, 7.66, N, 16.50.

2-methyl-*N*-((1-methyl-1H-imidazol-4-yl)methyl)-5,6,7,8-tetrahydroquinolin-8-amine **4b**: pale orange solid. Yield 67%. m.p. 90.3–92.2 °C. ^1^H NMR (CDCl_3_, 300 MHz) δ: 7.35 (s, 1H, H_12_), 7.26 (d, *J* = 7.6 Hz, 1H, H_3_), 6.88 (m, 2H, H_2_ + H_10_), 4. 16 (m, 3H, H_8_+H_7_), 3.61 (s, 3H, H_11_), 2.70 (m, 2H, H_4_), 2.46 (s, 3H, H_1_), 2.14 (m, 1H, H_5_), 2.01 (m, 1H, H_5_), 1.82 (m, 1H, H_6_), 1.68 (m, 1H, H_6_) ppm. ^13^C NMR (75 MHz, CDCl_3_) δ 155.28, 141.40, 137.26, 129.09, 121.45, 117.43, 77.52, 77.10, 76.67, 56.91, 44.80, 33.28, 29.64, 28.45, 28.34, 23.88, 19.53 ppm; FTIR (NaCl) v = 3369.17, 2929.65, 2859.59, 2386.02, 2230.33, 1660.12, 1596.42, 1572.80, 1509.59, 1470.96, 1449.80, 1259.37, 1198.51, 1099.24, 817.86, 730.95 cm^−1^; MS (ESI) for C_15_H_20_N_4_ *m/z* 257.28 [M + H]^+^. Anal. Calcd for C_15_H_20_N_4_: C, 70.28; H, 7.86; N, 21.86. Found: C, 70.54; H, 7.95, N, 21.75.

*N*-((1H-imidazol-2-yl)methyl)-2-methyl-5,6,7,8-tetrahydroquinolin-8-amine **5b**: orange solid. Yield 65%. m.p. 86.7–88.1 °C. ^1^H NMR (CDCl_3_, 300 MHz) δ: 7.34 (d, *J* = 6 Hz, 1H, H_2_), 7.26 (s, 1H, H_12_), 7.04 (s, 1H, H_10_), 7.02 (d, *J* = 6 Hz, 1H,H_3_), 4.26 (s, 2H, H_8_), 3.82 (t, *J* = 6 Hz, 1H, H_7_), 2.70 (m, 2H, H_6_), 2.45 (s, 3H, H_1_), 1.94 (m, 4H, H_4_ + H_5_) ppm. ^13^C NMR (75 MHz, CDCl_3_) δ 153.88, 140.32, 135.22, 128.89, 120.52, 118.13, 78.24, 76.01, 74.79, 54.12, 43.55, 31.24, 28.64, 28.56, 25.44, 20.39 ppm; FTIR (NaCl) v = 3358.71, 2918.55, 2833.42, 2368.21, 2229.38, 1663.52, 1598.55, 1570.18, 1500.01, 1463.78, 1441.81, 1251.71, 1059.48, 819.56, 732.51 cm^−1^; MS (ESI) for C_14_H_18_N_4_ *m/z* 243.17 [M + H]^+^. Anal. Calcd for C_14_H_18_N_4_: C, 69.39; H, 7.49; N, 23.12. Found: C, 69.64; H, 7.57, N, 22.96.

9-(((2-methyl-5,6,7,8-tetrahydroquinolin-8-yl)amino)methyl)-2,3,6,7-tetrahydro-1*H*,5*H*-pyrido[3 ,2,1-ij]quinolin-8-ol **6b**: orange solid. Yield 65%. m.p. 94.3–95.5 °C. ^1^H NMR (CDCl_3_, 300 MHz) δ: 7.26 (m, 1H, H_3_), 6.93 (d, *J* = 7.7 Hz, 1H, H_2_), 6.55 (d, *J* = 7.1 Hz, 1H, H_9_), 4.07 (d, *J* = 11.6 Hz, 1H, H_8_), 3.86 (d, *J* = 11.6 Hz, 1H, H_8_), 3.76 (m, 1H, H_7_), 3.06 (m, 2H, H_12_), 2.68 (m, 6H, H_4_ + H_13_ + H_15_), 2.45 (s, 3H, H_1_), 2.21 (m, 2H, H_10_), 1.95 (m, 4H, H_11_ + H_14_), 1.74 (m, 2H, H_5_), 1.68 (m, 2H, H_6_) ppm. ^13^C NMR (75 MHz, CDCl_3_) δ 155.43, 155.37, 153.77, 143.22, 137.23, 129.09, 125.41, 122.30, 112.16, 111.70, 109.17, 58.40, 50.35, 49.81, 29.71, 28.60, 28.33, 25.85, 24.10, 23.06, 21.92, 21.09, 20.41 ppm; FTIR (NaCl) v = 3284.71, 2961.47, 2925.61, 1943,15,1730.25, 1623.17, 1594.45, 1574.47, 1494.45, 1469.80, 1447.36, 1349.15, 1331.87, 1312.28, 1260.60, 1092.44, 1018.48, 880.69, 799.83, 731.44 cm^−1^; MS (ESI) for C_23_H_29_N_3_O *m/z* 363.96 [M + H]^+^. Anal. Calcd for C_23_H_29_N_3_O: C, 76.00; H, 8.04; N, 11.56. Found: C, 76.25; H, 8.12, N, 11.45.

Log *P_ow_* determination. RP-HPLC analyses were performed to correlate the hydrophobicity of the compounds with their retention time. The chromatograms were registered using a Partisil C18-ODS reversed-phase HPLC column, at 25 °C and with a water/acetonitrile ratio of 80/20 as mobile phase, and using KI as internal standard (flow rate of 1 mL/min, λ = 254 nm). The calibration curve was realized in comparison with reference compounds, chosen in commercially available compounds series (i.e., acetophenone, benzophenone, chalcone and cinnamic acid).

### 3.3. Cell Growth Assay

HeLa (human cervix carcinoma) and CEM (human T-lymphoblast) were grown in Dulbecco’s modified Eagle’s medium (DMEM; Gibco, Carlsbad, CA, USA), supplemented with 0.01 M Hepes (Gibco) and 1 mM sodium pyruvate (Gibco) in a humidified 5% CO_2_ incubator at 37 °C. HMEC-1 (human microvascular endothelial cells) was maintained in Dulbecco’s modified Eagle’s medium (DMEM)/low glucose containing glutamine (2 mM). The media were supplemented with 10% fetal bovine serum (FBS, Gibco), penicillin (50 g/mL), streptomycin (50 g/mL), and amphotericin B (1.25 g/mL). CEM cells were seeded in 96-well plates at 60,000 cells/well in the presence of different concentrations of the compounds, and allowed to proliferate for 72 h. HeLa cells were seeded in 96-well plates at 15,000 cells/well in the presence of different concentrations of the compounds. After 4 days of incubation, the cells were trypsinized and counted. HMEC-1 cells were seeded in 48-well plates at 20,000 cells/well. After 24 h, the compounds were added at different concentrations. The cells were allowed to proliferate for 4 days in the presence of the compounds, trypsinized, and counted. All cells were counted by means of a Coulter counter (Analis, Belgium).

A2780 (human ovarian carcinoma) and HT-29 (human colorectal adenocarcinoma) were grown in RPMI 1640 (Sigma Chemical Co., St. Louis, MO, USA), and MSTO-211H (human biphasic mesothelioma) were grown in RPMI 1640 (Sigma Chemical Co.) supplemented with 2.38 g/L Hepes, 0.11 g/L pyruvate sodium, and 2.5 g/L glucose. Then, 1.5 g/L NaHCO_3_, 10% heat-inactivated fetal calf serum (Biowest), 100 U/mL penicillin, 100 μg/mL streptomycin, and 0.25 μg/mL amphotericin B (Sigma Chemical Co.) were added to the media. The cells were cultured at 37 °C in a moist atmosphere of 5% carbon dioxide in air. Cells (2.5–3 × 10^4^) were seeded into each well of a 24-well cell culture plate. After incubation for 24 h, various concentrations of the test agents were added to the complete medium and incubated for a further 72 h. A Trypan blue assay was performed to determine cell viability. Cytotoxicity data were expressed as IC_50_ values, i.e., the concentration of the test compound able to induce a 50% reduction in cell number compared with control cultures.

### 3.4. Cell Cycle Analysis

A2780 cells (5 × 105) were seeded into a 6-well culture plate in complete medium. After incubation for 24 h, the test compound was added at the indicated concentration and cells were incubated for a further 28 h. After treatment, cells were harvested, centrifugated, and 300–500 × 10^3^ cells were fixed and permeabilized with 70% ice-cold ethanol at 4 °C for 20 min. The cells were washed twice with PBS and incubated in a staining solution containing 0.1 mg/mL RNAse and 36 μg/mL propidium iodide for 20 min at room temperature. The DNA content analysis was performed using a FACSCanto II flow cytometer (Becton-Dickinson, Mountain View, CA, USA).

### 3.5. Evaluation of Mitochondrial Transmembrane Potential

The mitochondrial transmembrane potential was evaluated by using the BD™ MitoScreen Kit (BD Pharmigen), based on the membrane-permeable lipophilic cationic fluorochrome 5,5′,6,6′-tetrachloro-1,1′,3,3′ tetraethylbenzimidazolcarbocyanine iodide (JC-1). The human ovarian carcinoma cell line (A2780) (5 × 10^5^) was seeded into each well in 6-well culture plates. After 24 h of incubation, the test agent was added to the complete medium at the indicated concentration and cells were incubated for a further 40 h. After treatment, cells were harvested, centrifuged, resuspended in JC-1 working solution, and incubated in the dark for 30 min at 37 °C in a CO_2_ incubator. Following incubation, cells were washed twice in phosphate-buffered saline (PBS), suspended in assay buffer, and promptly analyzed by a FACSCanto II flow cytometer.

### 3.6. Measurement of Reactive Oxygen Species (ROS)

The intracellular production of ROS was determined by using the cell-permeable fluorogenic probe 2′,7′-dichlorodihydrofluorescein diacetate (H_2_DCF-DA). A2780 cells (10^4^) were seeded in 96-well plates and incubated for 24 h. The medium was then removed, and the cells washed with PBS and incubated with 10 μM H_2_DCF-DA in the dark for 20 min at 37 °C and 5% carbon dioxide in air. Afterwards, the medium was removed, cells washed with PBS, and PBS/10 mM glucose containing the test compound was added. ROS production was estimated by monitoring the fluorescence increase of the probe (λex = 480, λem = 530) using a PerkinElmer VICTOR X3 plate reader.

## 4. Conclusions

The synthesis of six Schiff bases based on the tetrahydroquinoline scaffold and their corresponding amines was described, and the compounds were fully characterized. The antiproliferative activity of all the compounds was evaluated on both non-tumor and tumor cells. For compounds **3a**, **5a**, and **2b**, an enantioselective synthesis was performed to obtain them in an enantiopure form with the aim to shed light on the differences in the antiproliferative activity of the pure enantiomers. In order to achieve this goal, the enantiomers of the selected compounds were tested on three human tumor cell lines (HT-29, A2780, and MSTO-211H). The presence of a chiral center in compound **5a** appeared to be significant for its biological activity, with the ***R*** enantiomer displaying about a 3-fold higher inhibitory effect on the A2780 cell line with respect to its counterpart (***S***)-**5a.** Moreover, only the *R* enantiomer proved to be effective on the MSTO-211H cell line. A2780 cells exposed to (***R***)-**5a** showed substantial changes on the cell cycle progression and the study on the effect on the mitochondrial transmembrane potential highlighted the ability of **(*R*)-5a** to cause a significant depolarization of the mitochondrial membrane. Finally, the ability of **(*R*)-5a** to increase the levels of the ROS production in A2780 cells suggests the occurrence of intracellular oxidative damage, likely mitochondria mediated, that could contribute to its cytotoxic effect. In conclusion, compound **(*R*)-5a**, even in an average level, highlights the importance of chirality in the design and the development of new drug candidates endowed with promising antiproliferative activity.

## Figures and Tables

**Table 1:** Antiproliferative activity of compounds **1a**–**6a** and **1b**–**6b** in comparison with combretastatin A4P (CA-4P)**.**

Compound	IC_50_ (µM)		
	CEM	HeLa	HMEC-1
**1a**	>100	>100	>100
**2a**	>100	>100	>100
**3a**	33 ± 7	34 ± 6	77 ± 32
**4a**	>100	>100	>100
**5a**	43 ± 7	37 ± 9	57 ± 4
**6a**	64 ± 32	>100	82 ± 4
**1b**	66 ± 47	93 ± 10	63 ± 2
**2b**	23 ± 4	75 ± 11	60 ± 2
**3b**	>100	>100	>100
**4b**	>100	>100	89 ± 2
**5b**	51 ± 13	71 ± 3	53 ± 0
**6b**	77 ± 32	>100	81 ± 26
**CA-4P ^a^**	0.095 ± 0.006	0.079 ± 0.003	0.0029 ± 0

^a^ taken from [41].

**Table 2:** Antiproliferative activity of compounds **1a**–**6a** and **1b**–**6b** on three human tumor cell lines. Meta-amsacrine (*m*-AMSA) was reported as the reference.

Compound	IC_50_ (µM)		
	HT-29	A2780	MSTO-211H
**1a**	83 ± 5	65 ± 9	69 ± 3
**2a**	>100	>100	95 ± 5
**3a**	42 ± 6	16 ± 2	30 ± 8
**4a**	42 ± 8	11 ± 2	14 ± 1
**5a**	70 ± 2	13 ± 3	31 ± 7
**6a**	46 ± 7	36 ± 4	25 ± 11
**1b**	57 ± 6	42 ± 5	28 ± 4
**2b**	62 ± 8	25 ± 5	35 ± 10
**3b**	42 ± 6	11 ± 3	15 ± 3
**4b**	>100	>100	>100
**5b**	73 ± 6	38 ± 7	44 ± 5
**6b**	74 ± 3	36 ± 4	77 ± 7
***m*-AMSA ^a^**	0.039 ± 0.004	0.027 ± 0.005 ^a^	0.019 ± 0.001 ^a^

^a^ taken from [43].

**Table 3:** Antiproliferative activity of compounds **3a**, **5a**, and **2b** in chiral forms.

Compound	IC_50_ (µM)		
	HT-29	A2780	MSTO-211H
**(*R*)-3a**	>20	11.7 ± 2	14.9 ± 1.4
**(*S*)-3a**	>20	11.4 ± 0.4	11.8 ± 2.3
**(*R*)-5a**	>20	5.4 ± 1.3	15.1 ± 1.5
**(*S*)-5a**	>20	17.2 ± 3	>20
**(*R*)-2b**	>20	15.2 ± 2.3	>20
**(*S*)-2b**	>20	11.5 ± 2.6	>20

## Supplementary Materials

The following are available online. Figure S1: Effect of compound **(*R*)-5a** on the electrophoretic mobility of supercoiled DNA. Figure S2: Effect of compound **(*R*)-5a** on the relaxation of supercoiled pBR322 DNA by human recombinant topoisomerase I or II, Figure S3: Source of chirality: 2-methyl-5,6,7,8-tetrahydroquinolin-8-amine.
