# Supplementary material for: Biological Properties of New Chiral 2-Methyl-5,6,7,8-tetrahydroquinolin-8-amine-based Compounds
Source: Molecules. 2020 Nov 27;25(23):5561. doi: 10.3390/molecules25235561 (PMC7729733; doi:10.3390/molecules25235561)
Supplement: Supplementary file 1 [file molecules-25-05561-s001.pdf]

SUPPORTING INFORMATION

Biological properties of new chiral 2-methyl-5,6,7,8-tetrahydroquinolin-8-amine based compounds

Giorgio Facchetti,[a*] Michael S. Christodoulou,[a] Lina Barragán Mendoza,[b,c] Federico Cusinato,[b] Lisa Dalla Via,[b*] Isabella Rimoldi[a]

[a] *DISFARM, Sezione di Chimica Generale e Organica "A. Marchesini" Università degli Studi di Milano via Venezian, 21, 20133, Milano, Italy*

[b] *Dipartimento di Scienze del Farmaco, Università degli Studi di Padova, via F. Marzolo, 5, 35131, Padova, Italy*

[c] *Facultad de Ciencias Químicas, Universidad de Colima, Carr. Colima-Coquimatlán km 9, Coquimatlán, Colima, 28400, México*

INDEX

Chiral purity of 2-methyl-5,6,7,8-tetrahydroquinolin-8-amine……………………………………………S1

Additional pharmacological experiments…………………………………..………………………S2

^{1}H and ^{13}C-NMR spectra and Log*P* of compounds………………………..……………………..….S4

Figure 1SI: Source of chirality: 2-methyl-5,6,7,8-tetrahydroquinolin-8-amine. The enantiomeric eccess by HPLC was evaluated after Boc protection step. Chiral column: Phonomenex Lux Cellulose-4; eluent hexane/isopropanol=90/10; λ=254 nm; flow=1.0 mL/min.

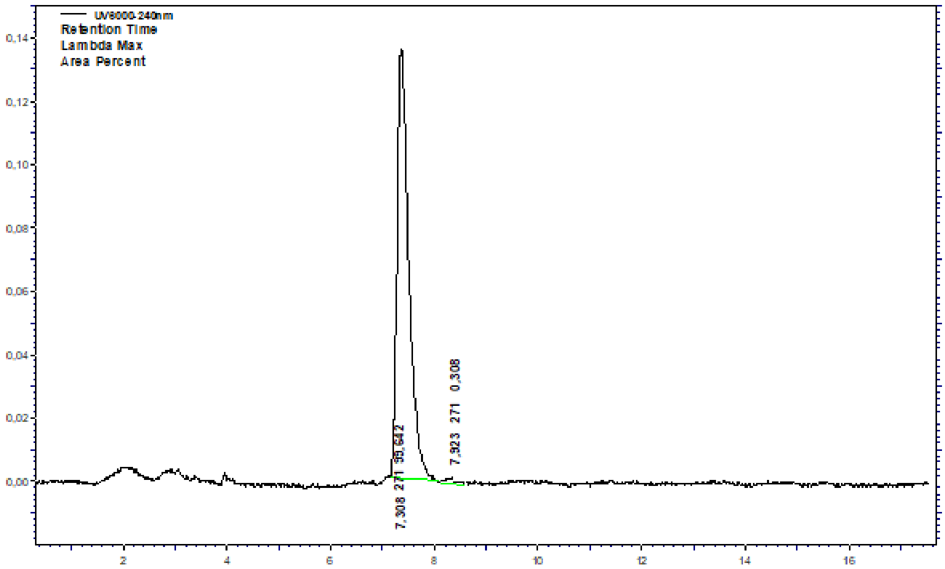

Retention time *R*-enantiomer: 7.3 min; 99.3 % e.e.

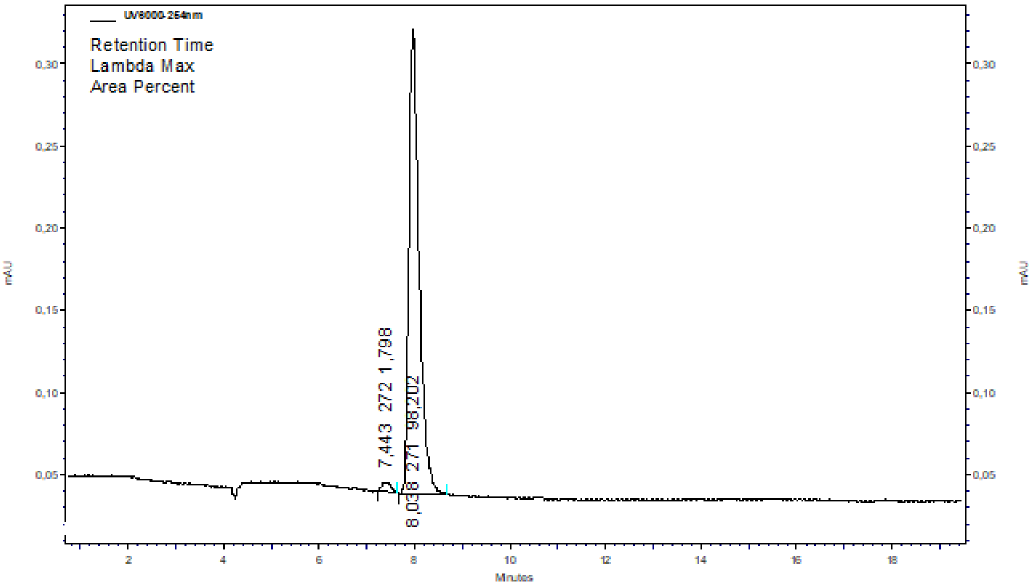

Retention time *S*-enantiomer: 8.0 min; 96.4 % e.e.

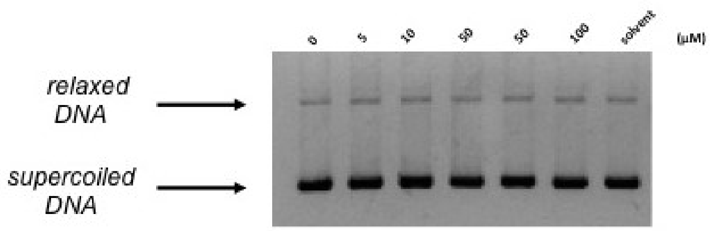

Figure 2SI. Effect of compound **(*R*)-5a** on the electrophoretic mobility of supercoiled DNA. Supercoiled plasmid pBR322 DNA was incubated in the absence or in the presence of **(*R*)-5a** at indicated concentrations. The effect of solvent alone on DNA was also assayed (solvent).

Unwinding assay

Supercoiled pBR322 plasmid DNA (0.15 µg, Fermentas Life Sciences) was incubated in TAE buffer (0.04 M Tris, 0.02 M Acetic Acid, EDTA 1 mM, pH=8) with test compound at indicated concentrations for 3 h at 37 °C in 10 µL final volume. 3 µL of loading buffer (0.125% bromophenol blue, 0.125% xylene cyanol and 50% glycerol) was added to each sample and DNA was separated by electrophoresis on a 0.8% agarose gel at room temperature. The gels were stained with ethidium bromide (1 µg/mL) in TAE buffer, transilluminated by UV light, and the fluorescence emission was visualized by a CCD camera coupled to a Bio-Rad Gel Doc XR apparatus.

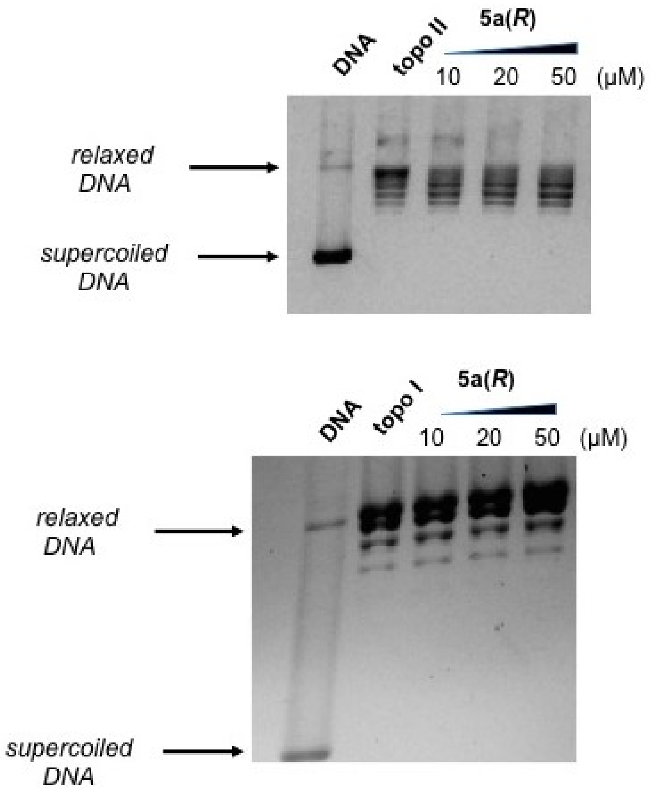

Figure 3SI. Effect of compound **(R)-5a** on the relaxation of supercoiled pBR322 DNA by human recombinant topoisomerase I or II. Supercoiled plasmid pBR322 DNA (DNA) was incubated with topo I (topo I) or II in the absence (topo II) and in the presence of **(R)-5a** at indicated concentrations.

Topoisomerase I and II relaxation assay

Supercoiled pBR322 plasmid DNA (20 ng, Fermentas Life Sciences) and 5U topoisomerase I (human topoisomerase I, TopoGEN) or alternatively, supercoiled pBR322 plasmid DNA (0.25 µg) and 1U topoisomerase II (human topoisomerase II alpha, Inspiralis) were incubated in the presence of test compound as indicated for 60 min at 37 °C in 20 µL reaction buffer.Reactions were stopped by adding 4 µL stop buffer (5% sodium dodecyl sulfate, 0.125% bromophenol blue, and 30% glycerol), 50 µg/mL proteinase K (Sigma) and incubating for a further 30 min at 37 °C. The samples were separated by electrophoresis on a 1% agarose gel at room temperature. The gels were stained with ethidium bromide 1 µg/mL in TAE buffer (0.04 M Tris-acetate and 0.001 M EDTA), transilluminated by UV light, and fluorescence emission was visualized by a CCD camera coupled to a Bio-Rad Gel Doc XR apparatus.

^{1}H- NMR in $CDCl_3$ (300 MHz) of (E)-2-methyl-6-(((2-methyl-5,6,7,8-tetrahydroquinolin-8-yl)imino)methyl)phenol **1a**

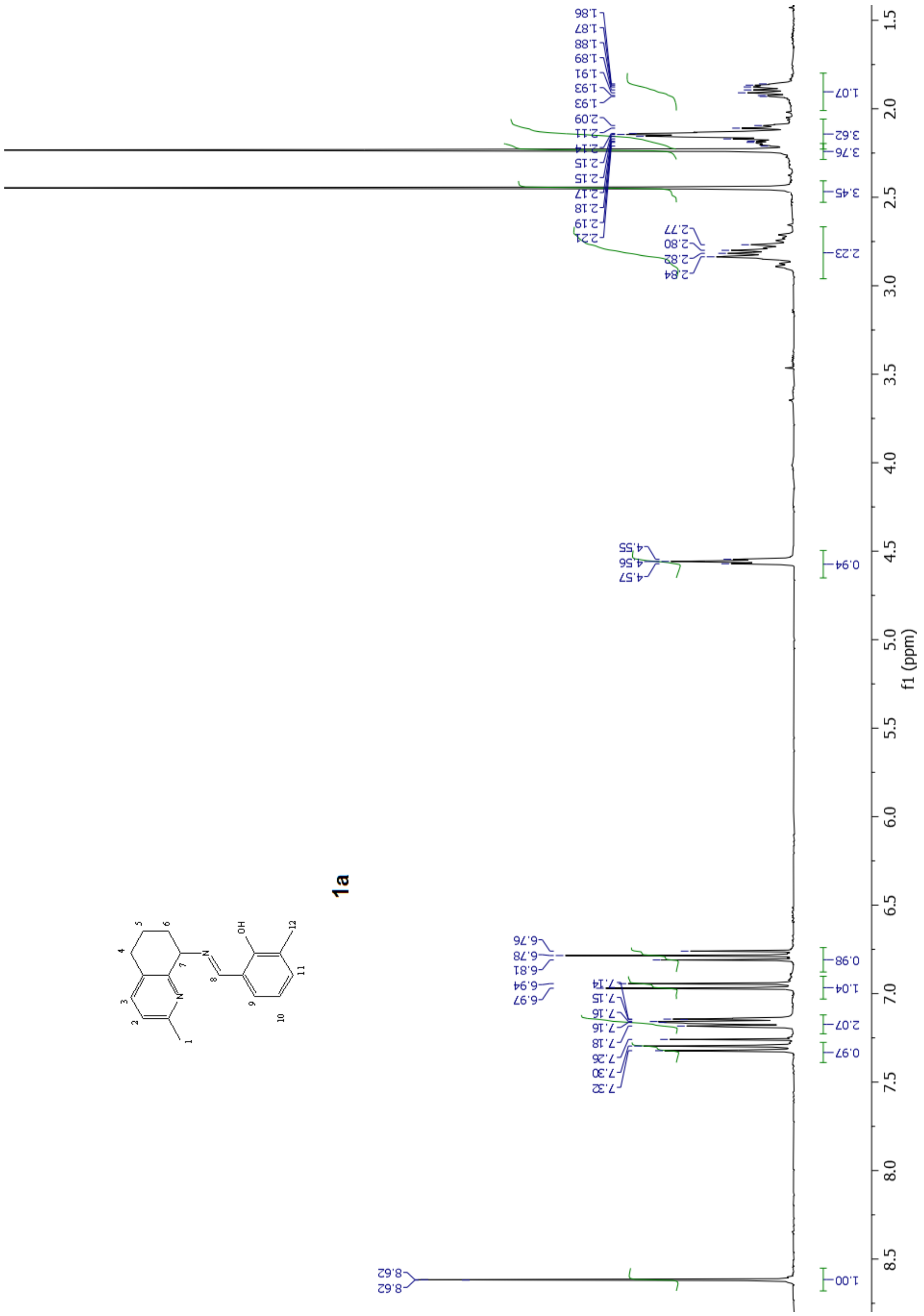

^{13}C- NMR in $CDCl_3$ (75 MHz) of (E)-2-methyl-6-(((2-methyl-5,6,7,8-tetrahydroquinolin-8-yl)imino)methyl)phenol **1a**

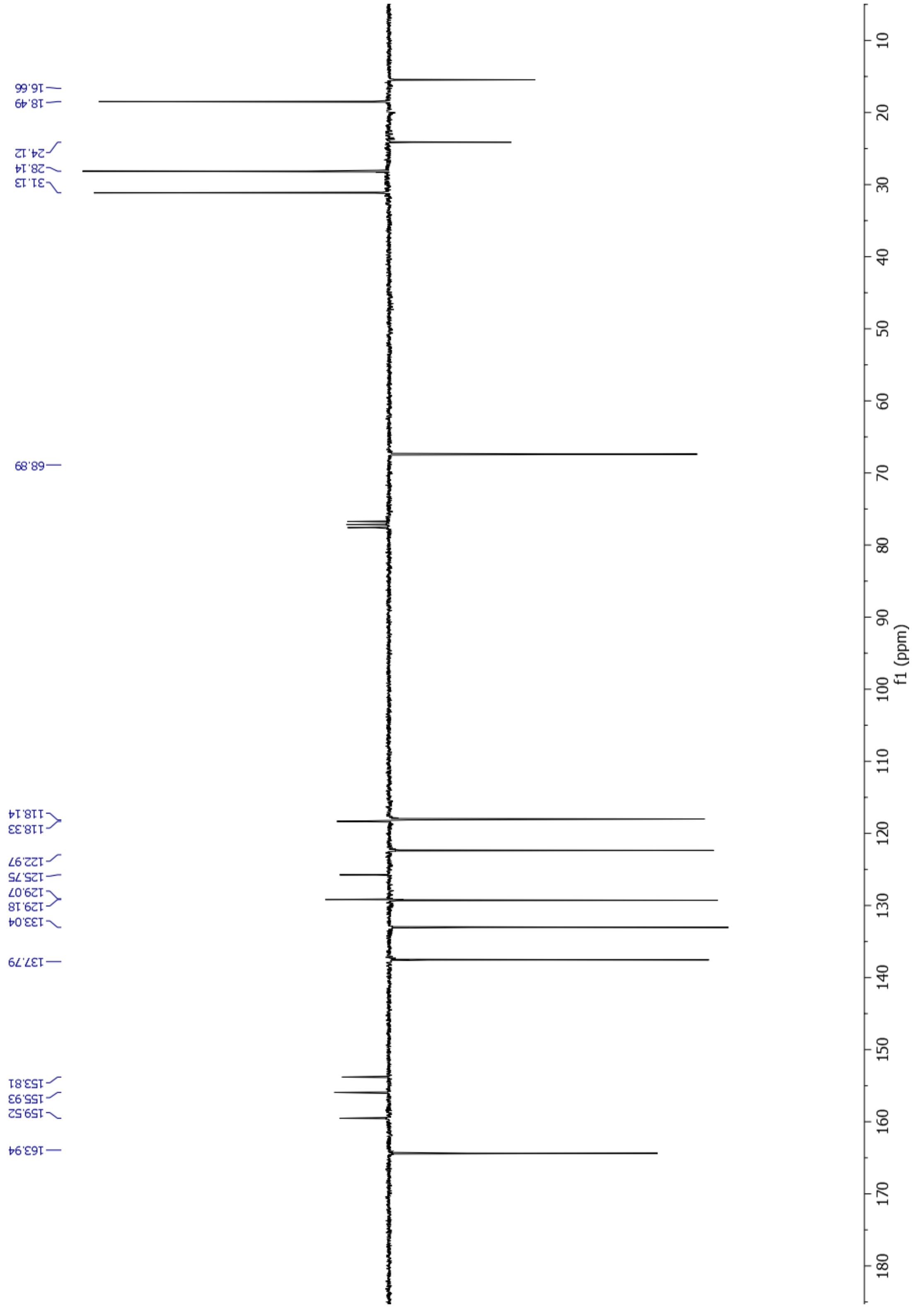

Log *P* of (E)-2-methyl-6-(((2-methyl-5,6,7,8-tetrahydroquinolin-8-yl)imino)methyl)phenol **1a**

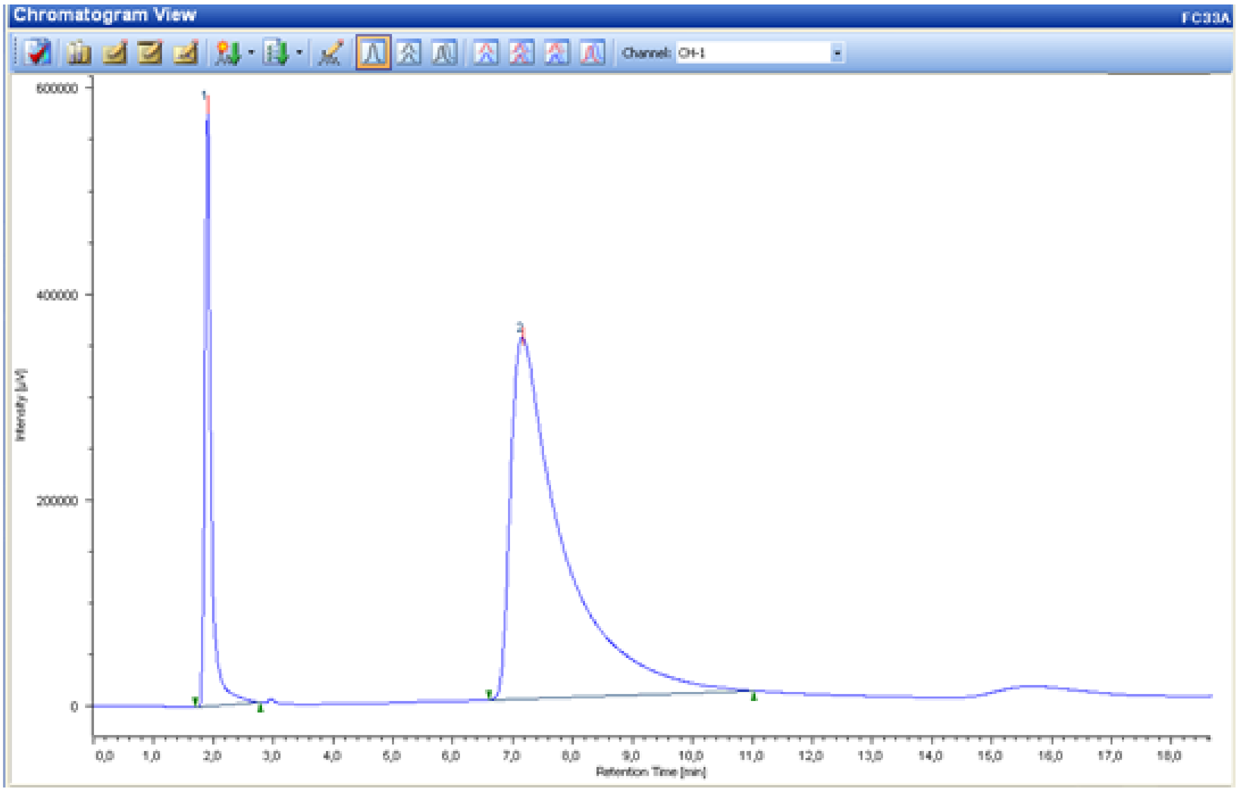

Peak Name	CH	tR	Area	Height	Area%	Height%	Quantity	NTP	Resolution	Symmetry Factor
Unknown	1	1,900	4251631	2581000	16,619	62,414	N/A	2259	7,126	1,604
Unknown	1	7,168	21330704	360415	83,301	37,606	N/A	471	N/A	3,716

Log $P_{o/w}$ = 3.73

^{1}H-NMR in $CDCl_3$ (300 MHz) of (*E*)-4-methyl-2-(((2-methyl-5,6,7,8-tetrahydroquinolin-8-yl)imino)methyl)phenol **2a**

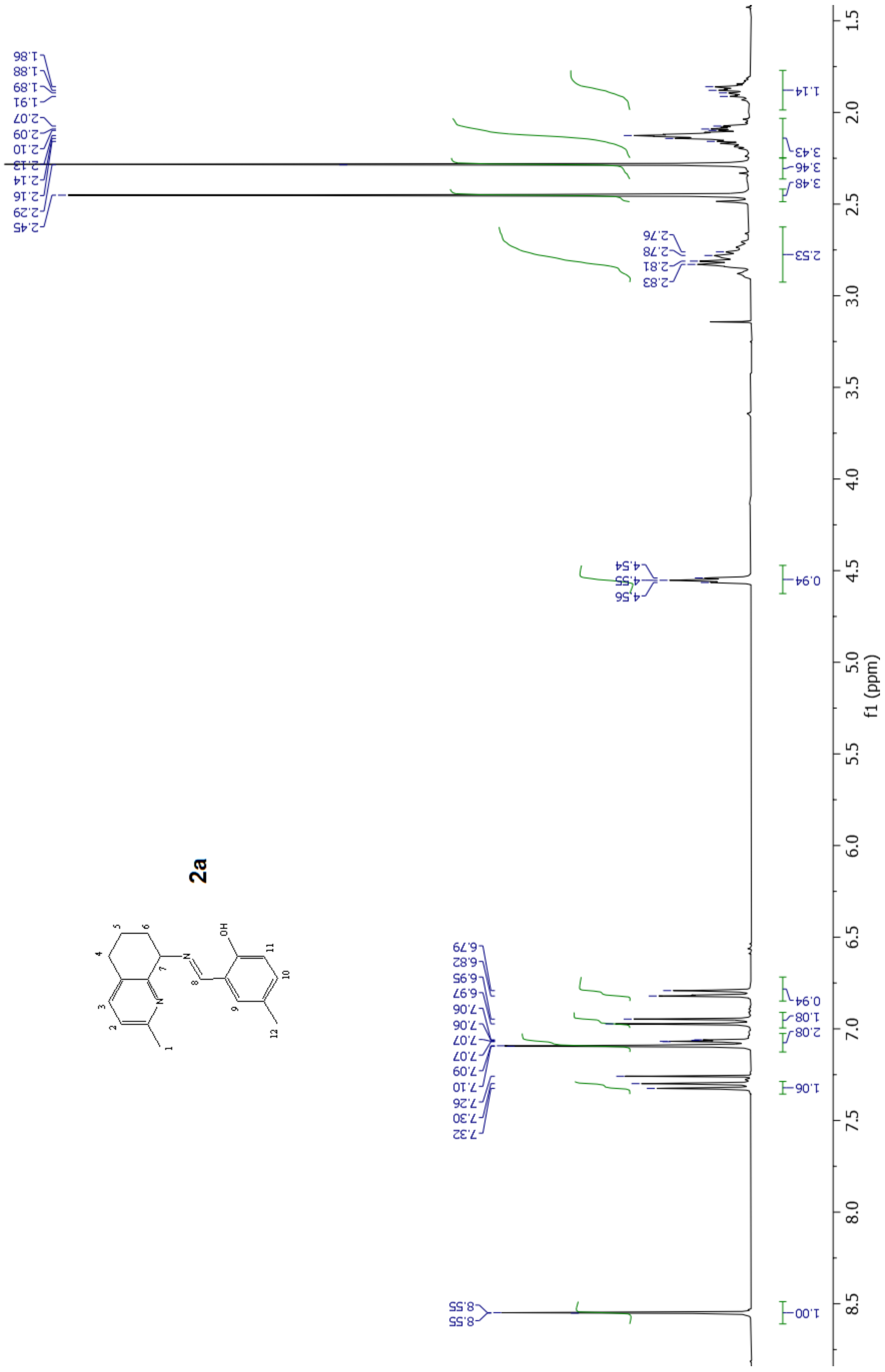

^{13}C-NMR in $CDCl_3$ (75 MHz) of (*E*)-4-methyl-2-(((2-methyl-5,6,7,8-tetrahydroquinolin-8-yl)imino)methyl)phenol **2a**

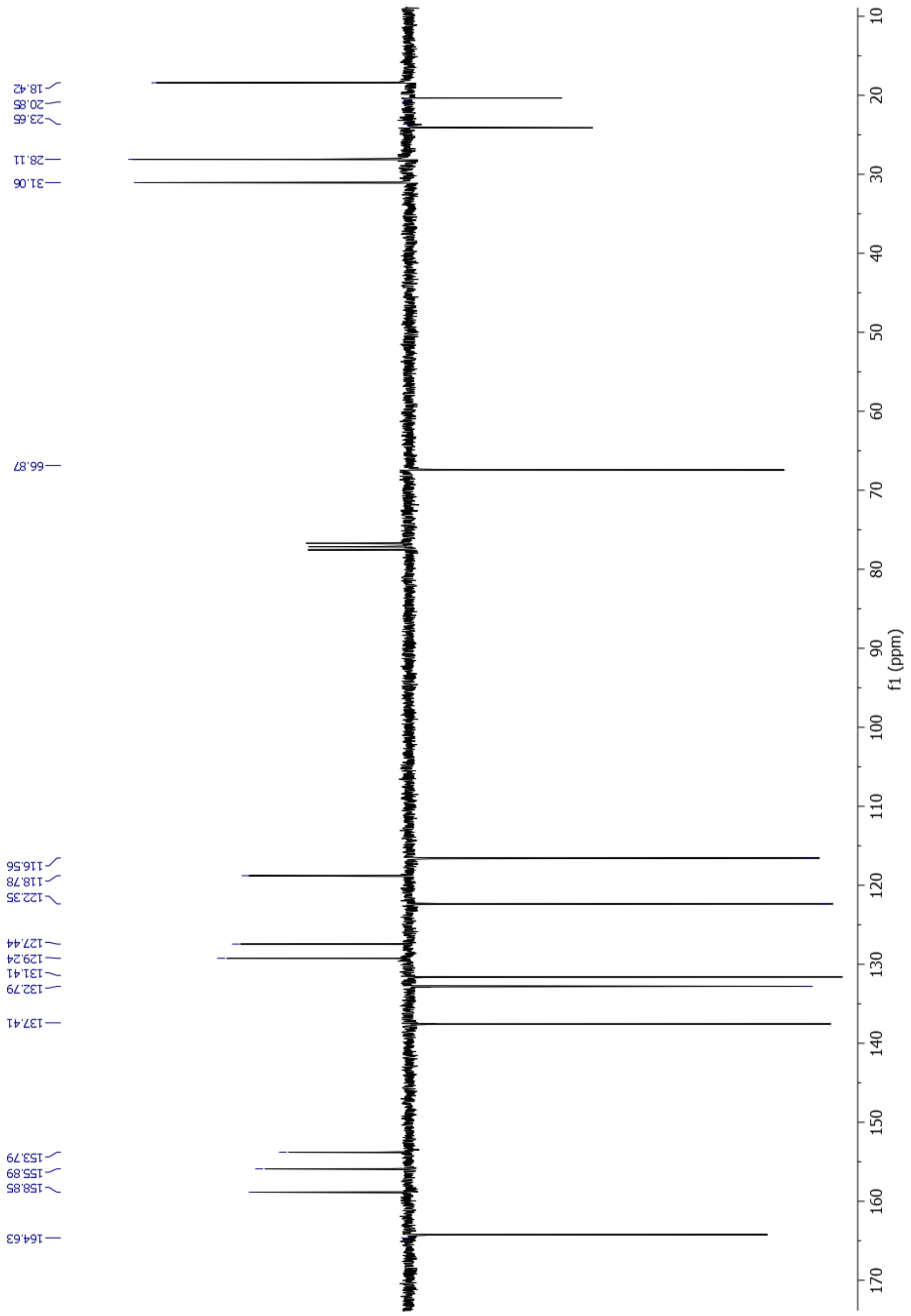

Log *P* of (*E*)-4-methyl-2-(((2-methyl-5,6,7,8-tetrahydroquinolin-8-yl)imino)methyl)phenol **2a**

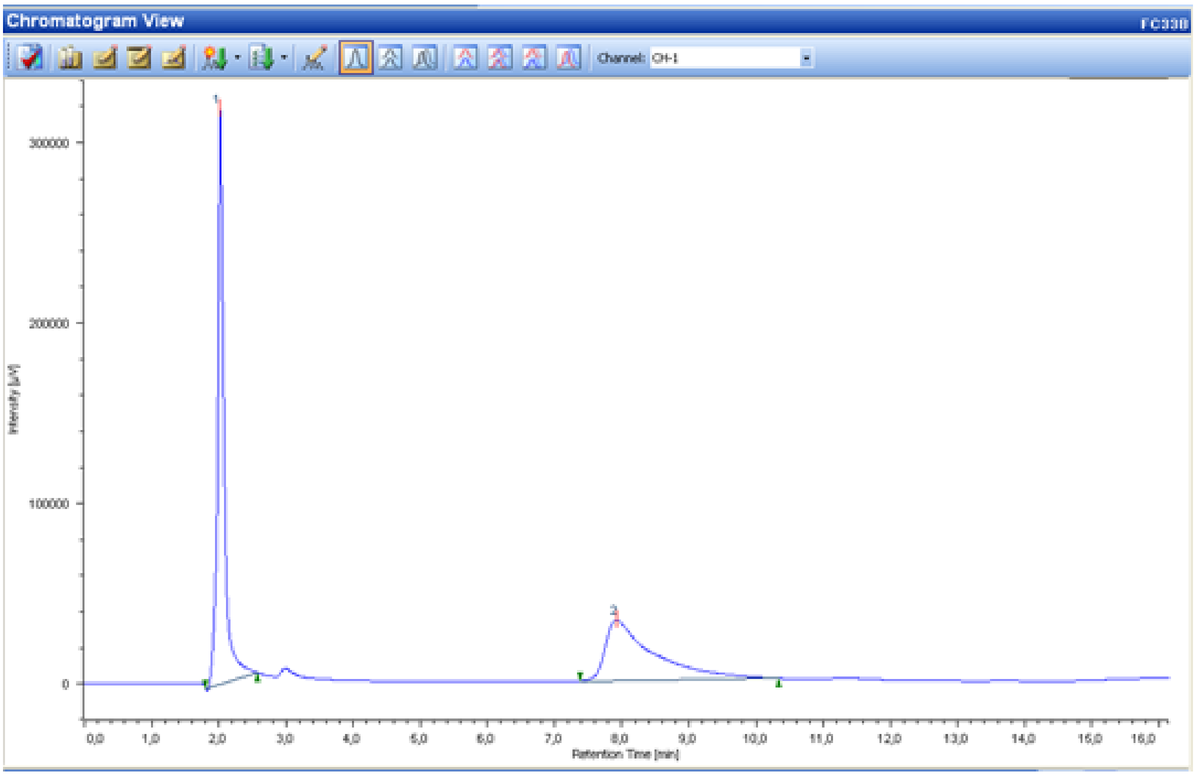

Peak Name	CH	tR	Area	Height	Area%	Height%	Quantity	NTP	Resolution	Symmetry Factor
Unknown	1	2,017	2130436	310411	66,923	90,457	N/A	3290	9,778	1,324
Unknown	1	7,917	1679124	33691	44,077	9,543	N/A	876	N/A	3,058

Log $P_{o/w}$ = 3.81

^{1}H-NMR in $CDCl_3$ (300 MHz) of (*E*)-N-(2-methyl-5,6,7,8-tetrahydroquinolin-8-yl)-1-(pyridin-2-yl)methanimine **3a**

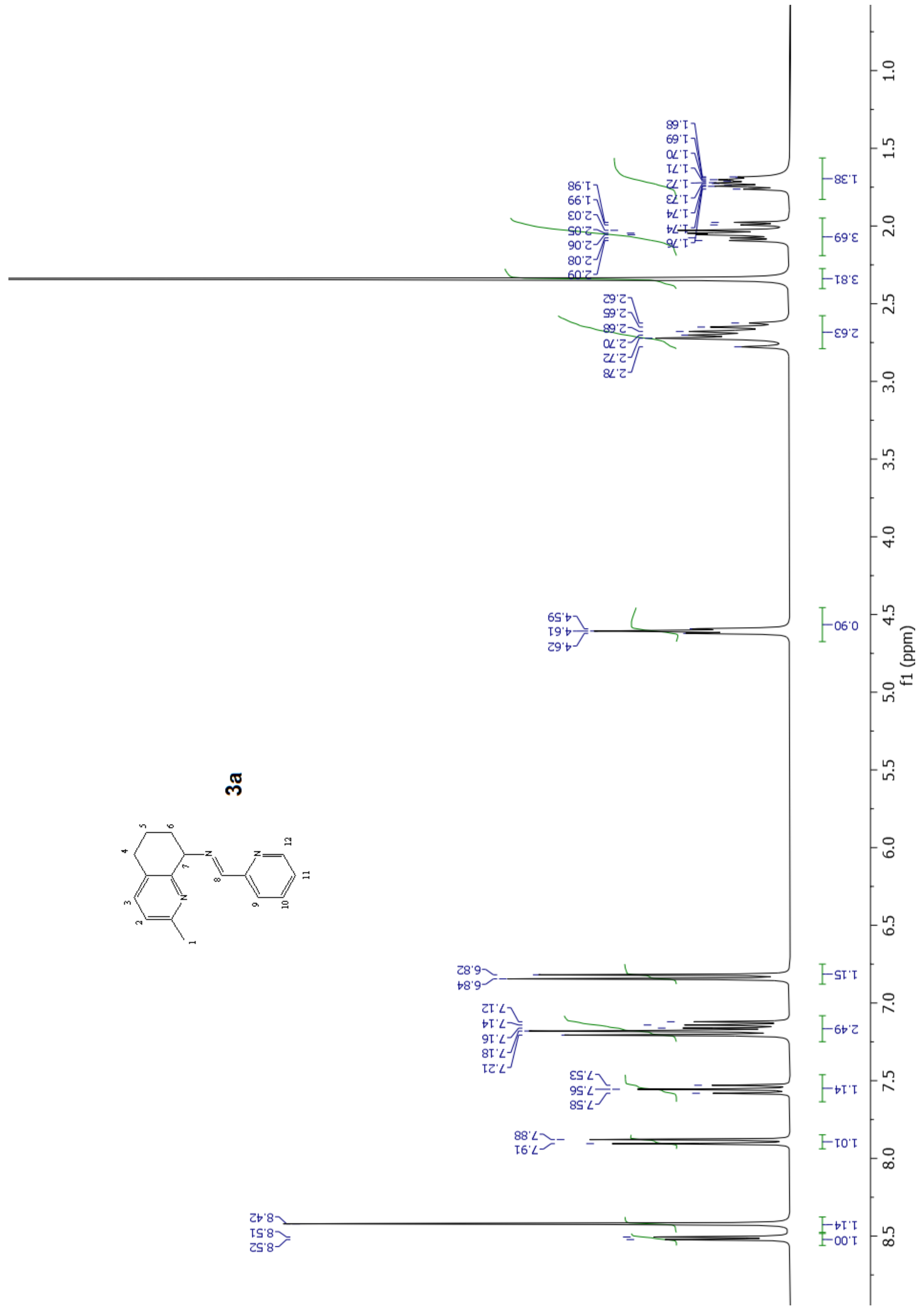

^{13}C-NMR in $CDCl_3$ (75 MHz) of (*E*)-N-(2-methyl-5,6,7,8-tetrahydroquinolin-8-yl)-1-(pyridin-2-yl)methanimine **3a**

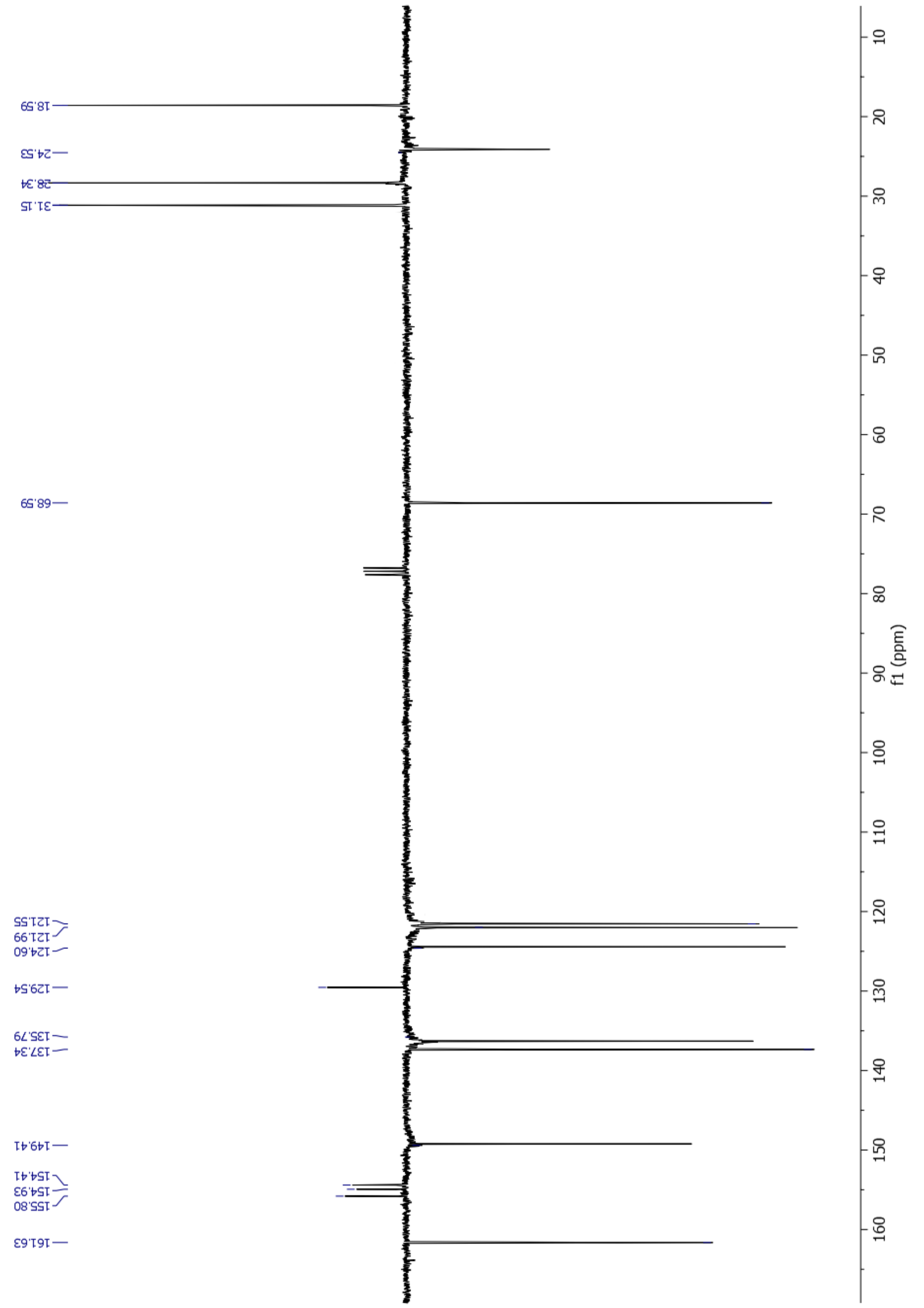

Log *P* of (*E*)-N-(2-methyl-5,6,7,8-tetrahydroquinolin-8-yl)-1-(pyridin-2-yl)methanimine **3a**

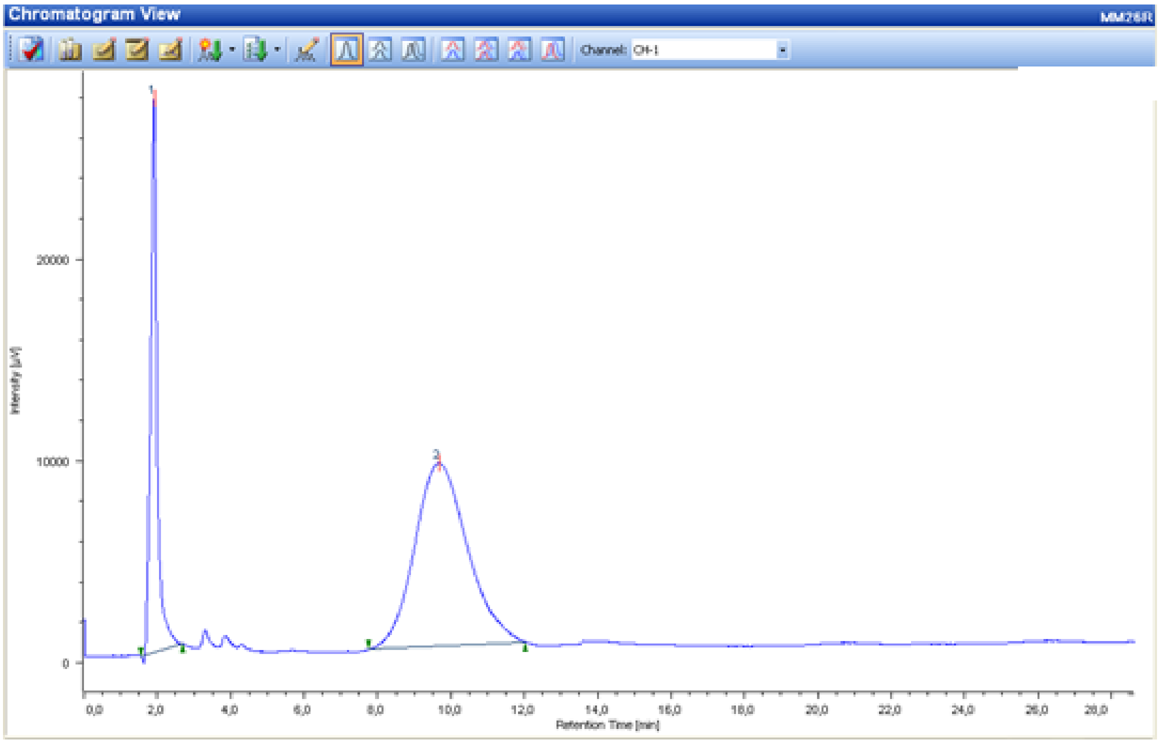

Peak Name	CH	tR	Area	Height	Area%	Height%	Quantity	NTP	Resolution	Symmetry Factor
Unknown	1	1,917	337959	27343	27,139	75,135	N/A	763	9,839	1,207
Unknown	1	9,985	907335	9049	72,861	24,865	N/A	608	N/A	1,151

Log $P_{o/w}$ = 4.07

^{1}H-NMR in $CDCl_3$ (300 MHz) of (*E*)-1-(1-methyl-1H-imidazol-4-yl)-N-(2-methyl-5,6,7,8-tetrahydroquinolin-8-yl)methanimine **4a**

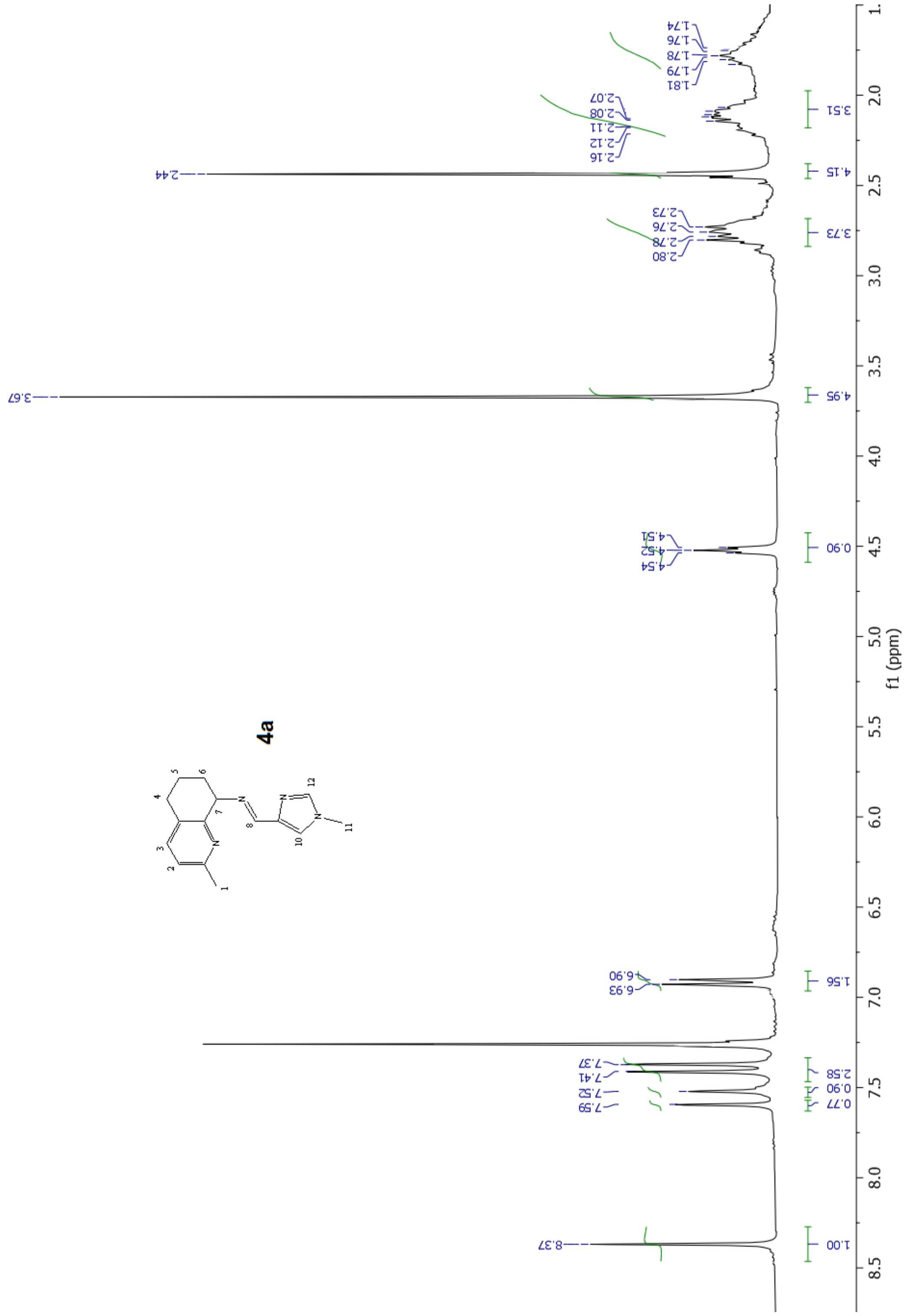

^{13}C-NMR in $CDCl_3$ (75 MHz) of (*E*)-1-(1-methyl-1H-imidazol-4-yl)-N-(2-methyl-5,6,7,8-tetrahydroquinolin-8-yl)methanimine **4a**

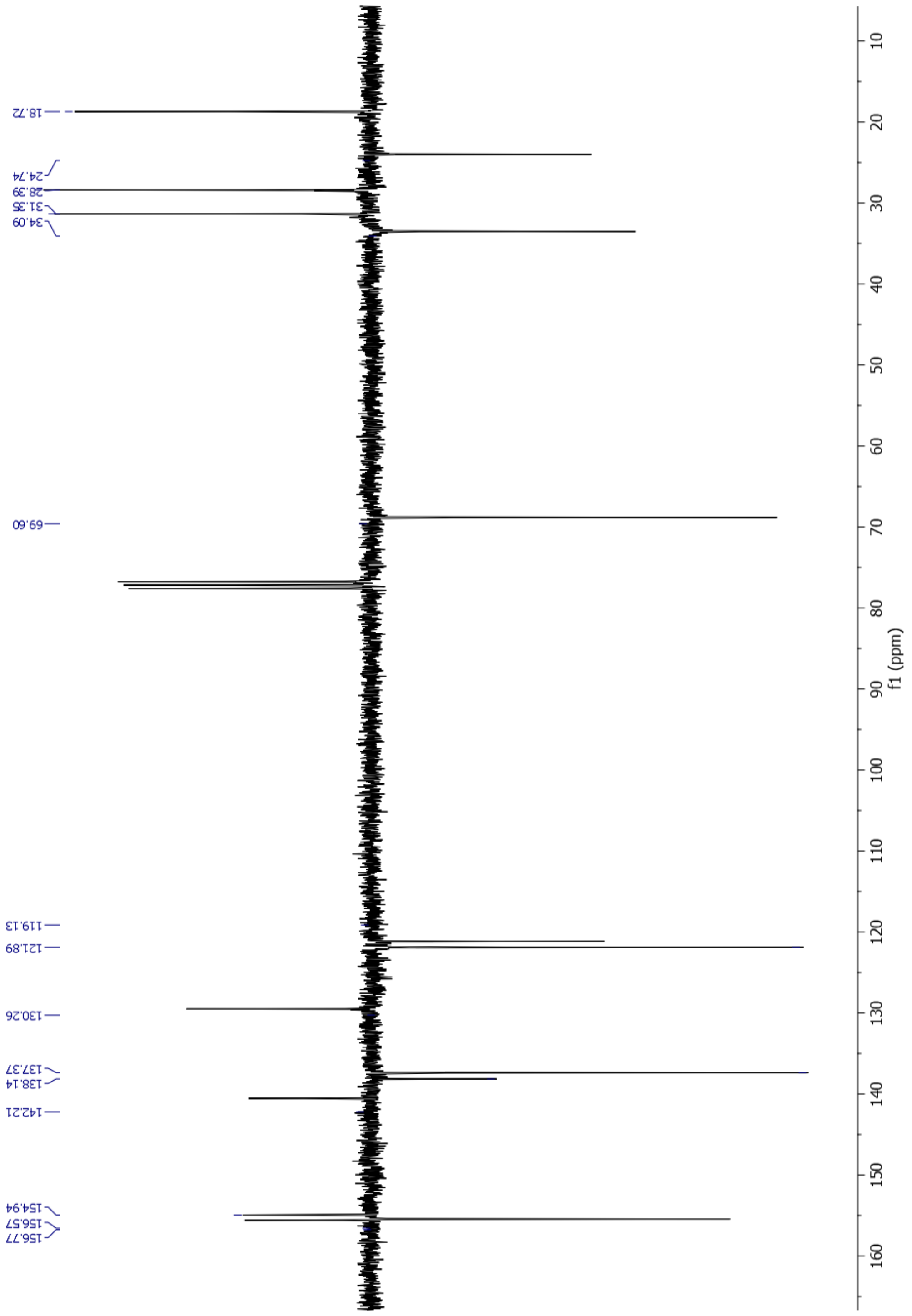

Log *P* of (*E*)-1-(1-methyl-1H-imidazol-4-yl)-N-(2-methyl-5,6,7,8-tetrahydroquinolin-8-yl)methanimine **4a**

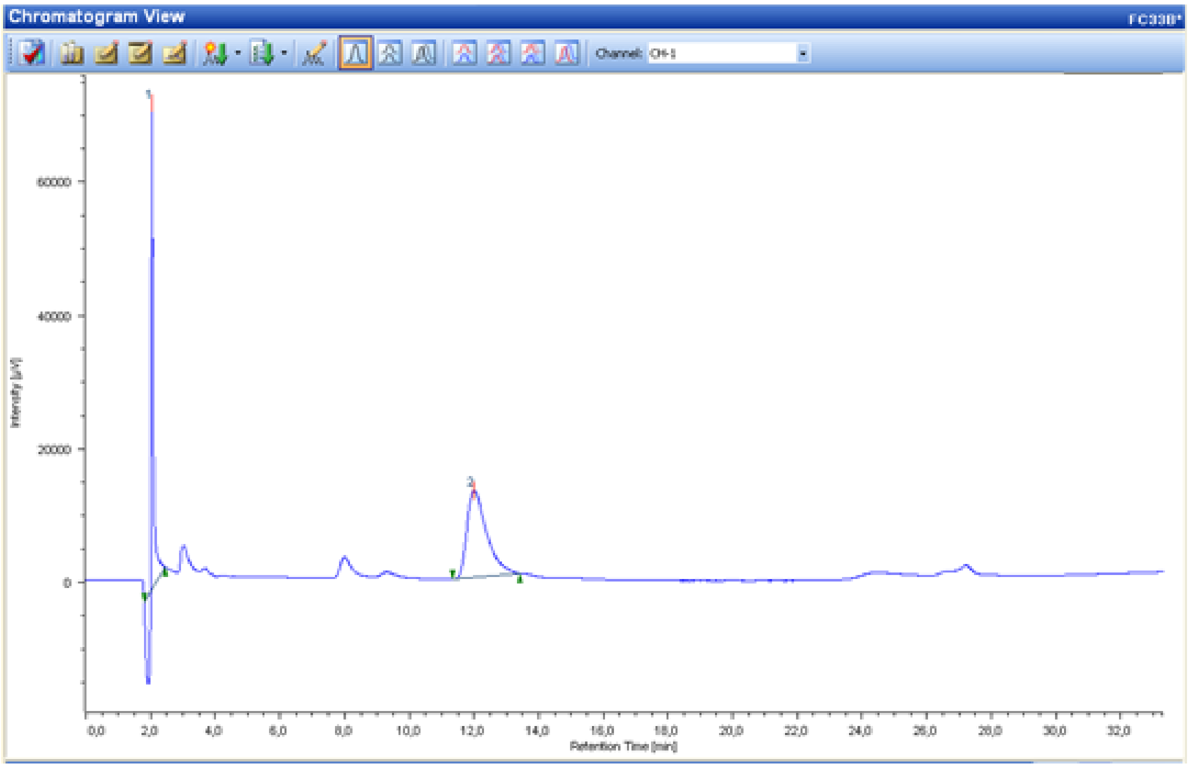

Peak Name	CH	tR	Area	Height	Area%	Height%	Quantity	NTP	Resolution	Symmetry Factor
Unknown	1	2,042	336400	72577	37,762	64,704	N/A	6623	16,962	2,545
Unknown	1	11,992	664430	13025	62,238	15,216	N/A	1907	N/A	1,741

Log $P_{o/w}$ = 3.94

^{1}H-NMR in $CDCl_3$ (300 MHz) of (*E*)-1-(1H-imidazol-2-yl)-N-(2-methyl-5,6,7,8-tetrahydroquinolin-8-yl)methanimine **5a**

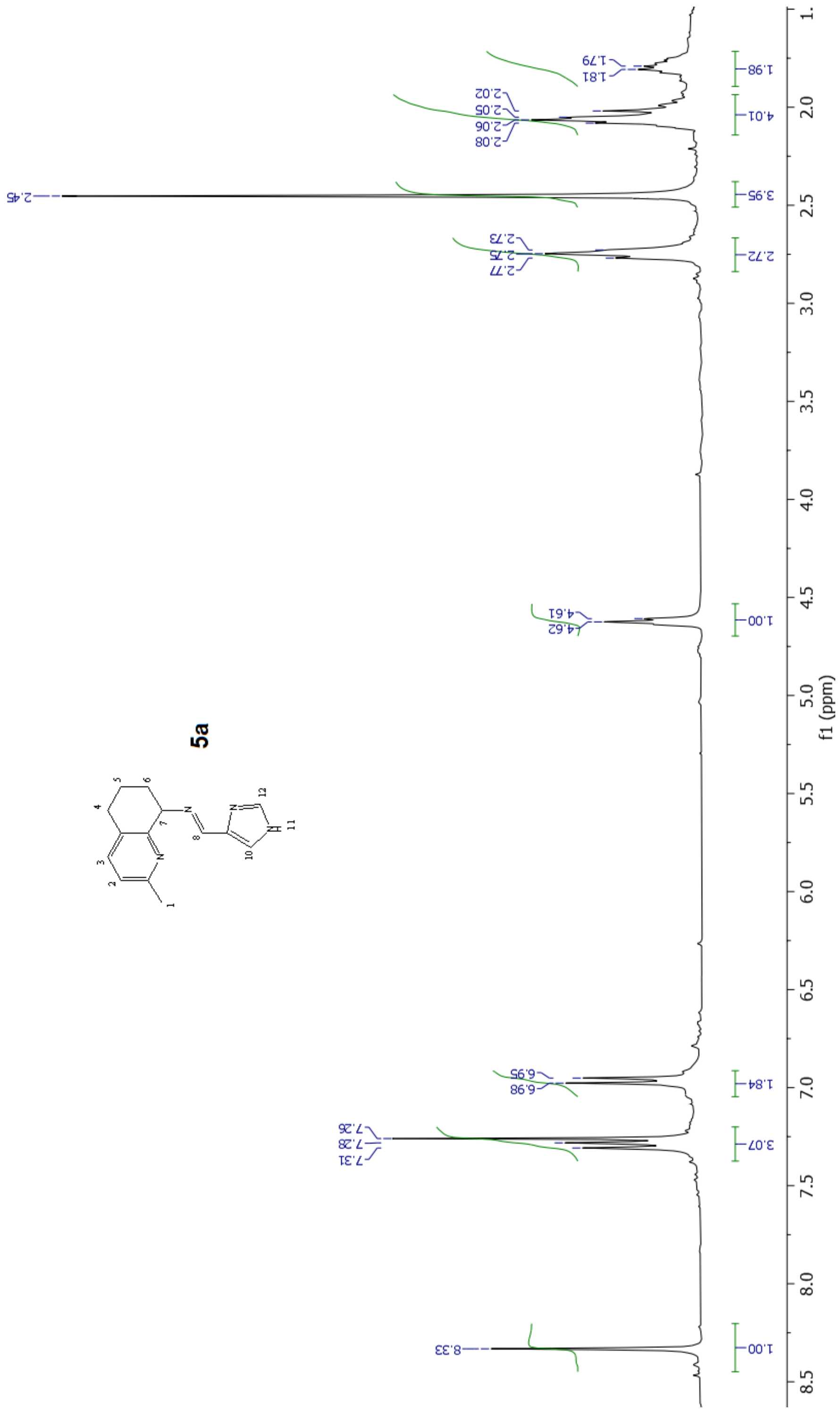

^{13}C-NMR in $CDCl_3$ (75 MHz) of (*E*)-1-(1H-imidazol-2-yl)-N-(2-methyl-5,6,7,8-tetrahydroquinolin-8-yl)methanimine **5a**

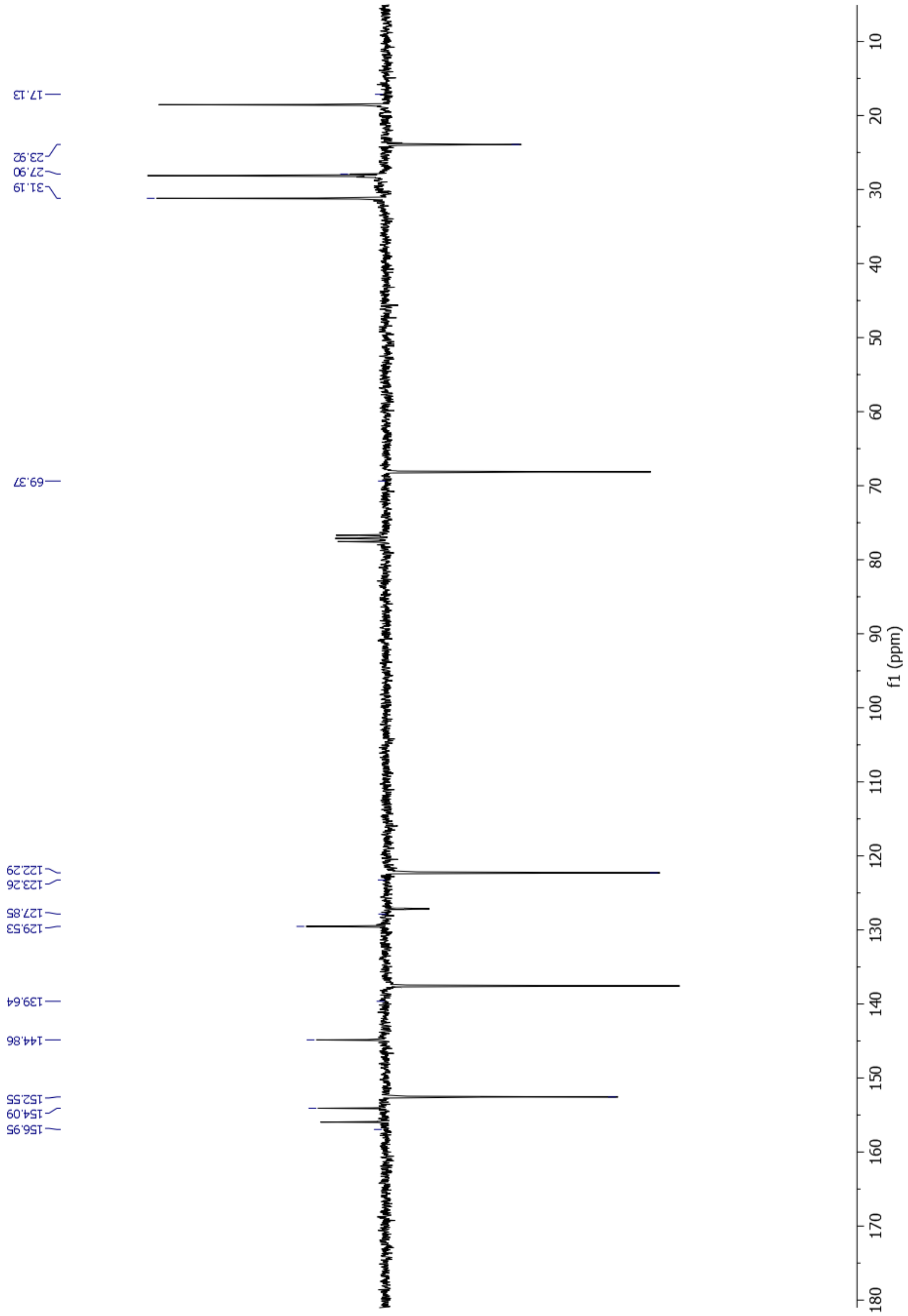

Log *P* of (*E*)-1-(1H-imidazol-2-yl)-N-(2-methyl-5,6,7,8-tetrahydroquinolin-8-yl)methanimine **5a**

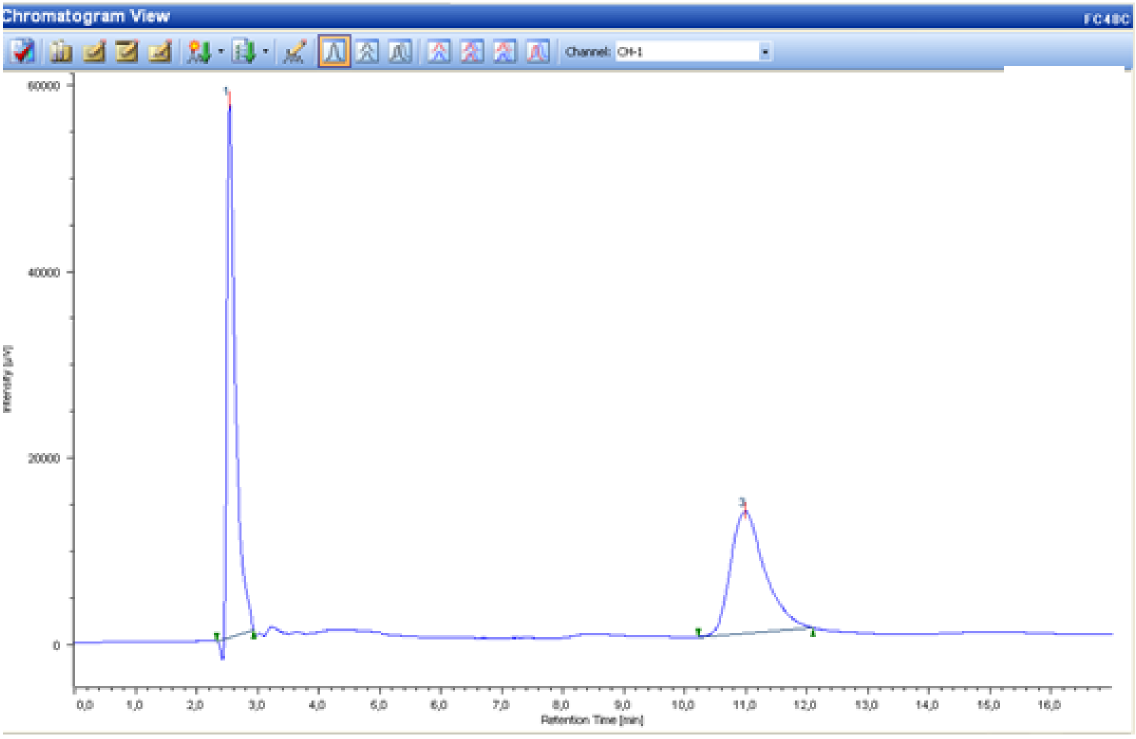

Peak Name	CH	tR	Area	Height	Area%	Height%	Quantity	NTP	Resolution	Symmetry Factor
Unknown	1	2,542	512946	67412	49,967	63,154	N/A	1906	6,515	2,268
Unknown	1	10,975	550006	13044	50,033	14,349	N/A	1968	N/A	1,419

Log $P_{o/w}$ = 4.30

^{1}H-NMR in $CDCl_3$ (300 MHz) of (*E*)-9-(((2-methyl-5,6,7,8-tetrahydroquinolin-8-yl)imino)methyl)-2,3,6,7-tetrahydro-1H,5H-pyrido [3,2,1-ij]quinolin-8-ol **6a**

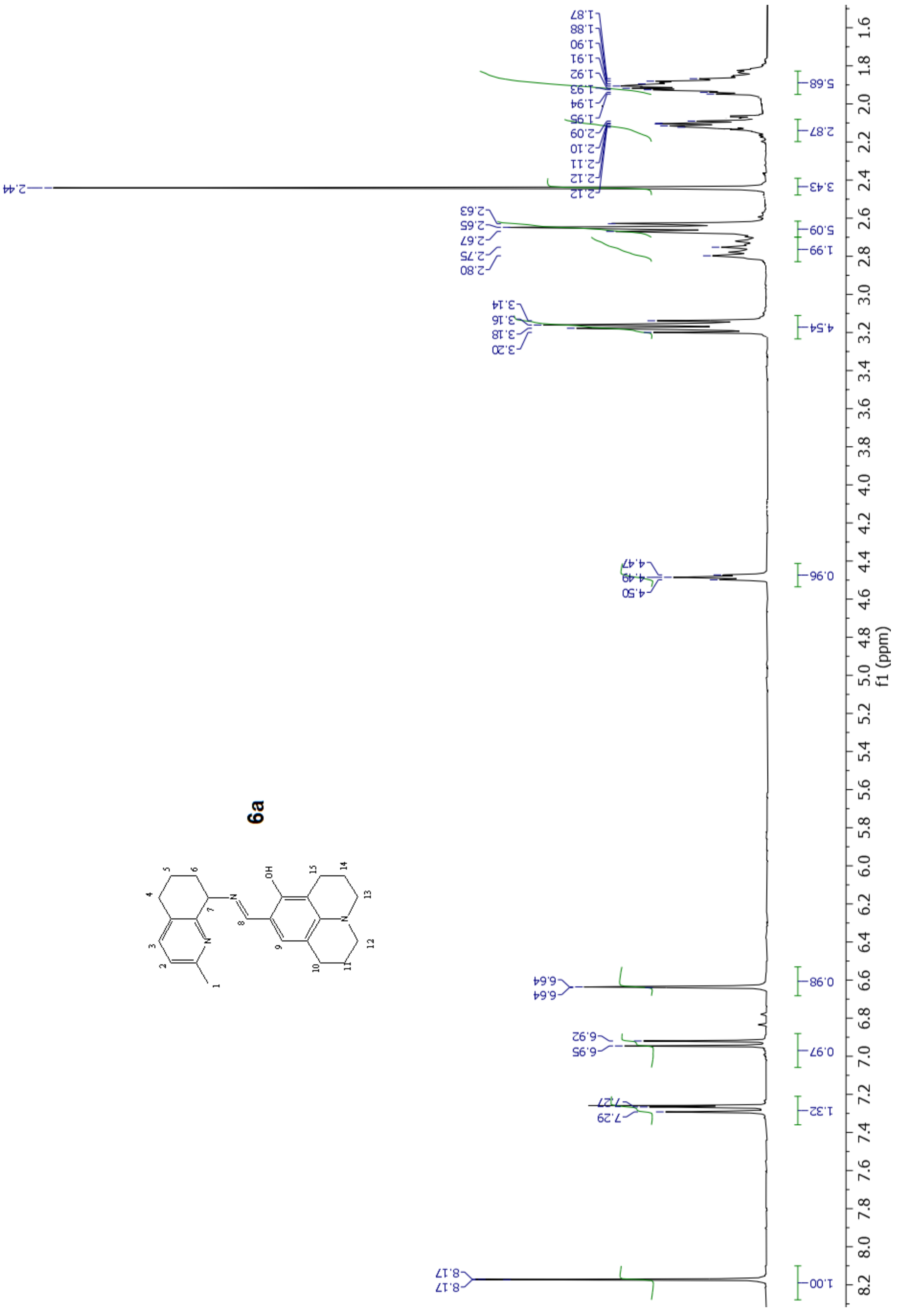

^{13}C-NMR in $CDCl_3$ (75 MHz) of (*E*)-9-(((2-methyl-5,6,7,8-tetrahydroquinolin-8-yl)imino)methyl)-2,3,6,7-tetrahydro-1H,5H-pyrido [3,2,1-ij]quinolin-8-ol **6a**

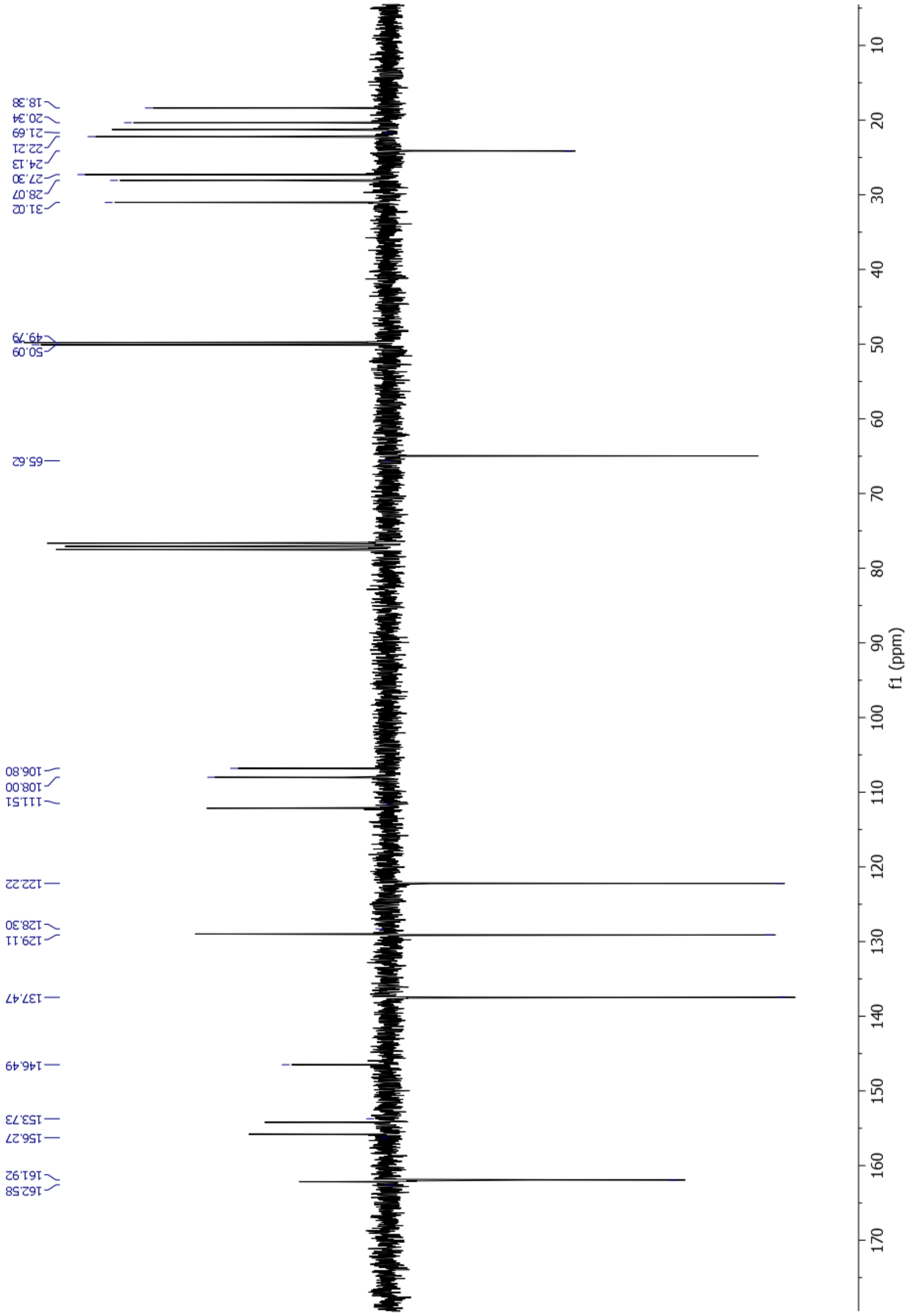

Log *P* of (*E*)-9-(((2-methyl-5,6,7,8-tetrahydroquinolin-8-yl)imino)methyl)-2,3,6,7-tetrahydro-1H,5H-pyrido [3,2,1-ij]quinolin-8-ol **6a**

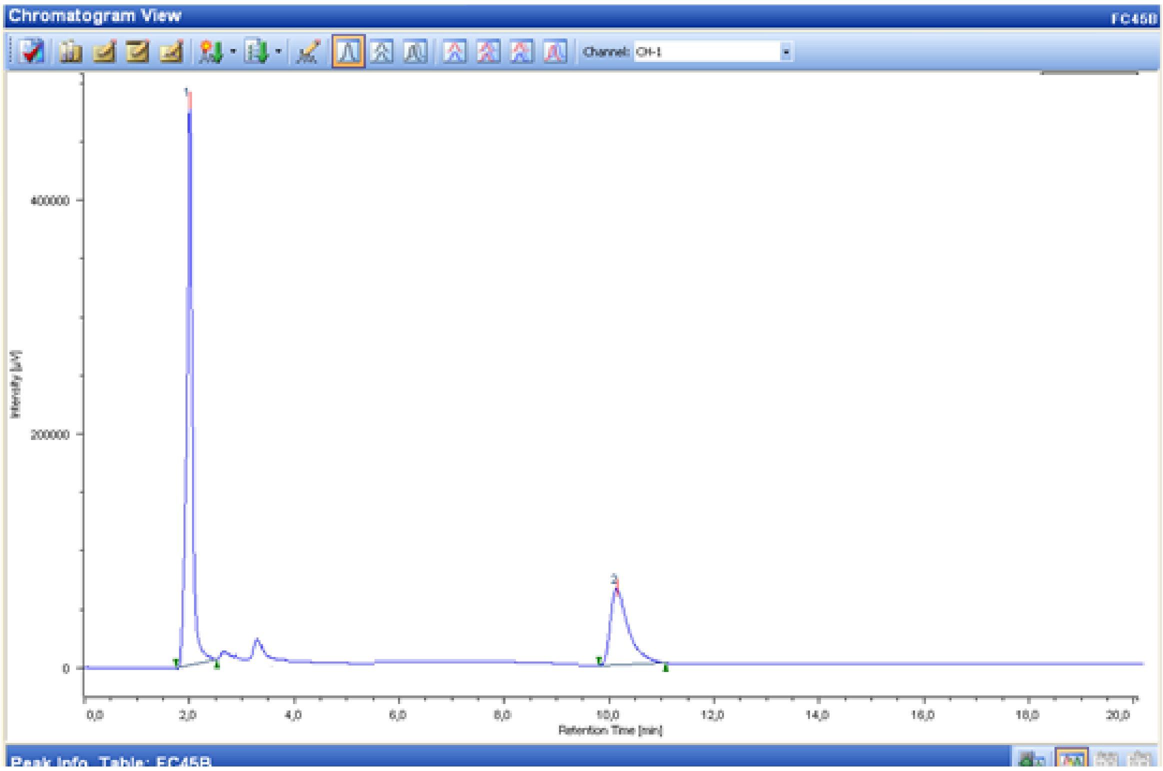

Peak Name	CH	tR	Area	Height	Area%	Height%	Quantity	NTP	Resolution	Symmetry Factor
Unknown	1	2,030	537444	36293	47,225	55,874	N/A	1530	5,442	1,848
Unknown	1	10,238	407785	11254	52,775	36,125	N/A	1326	N/A	1,730

Log $P_{o/w}$ = 4.09

^{1}H-NMR in $CDCl_3$ (300 MHz) of 2-methyl-6-(((2-methyl-5,6,7,8-tetrahydroquinolin-8-yl)amino)methyl)phenol **1b**

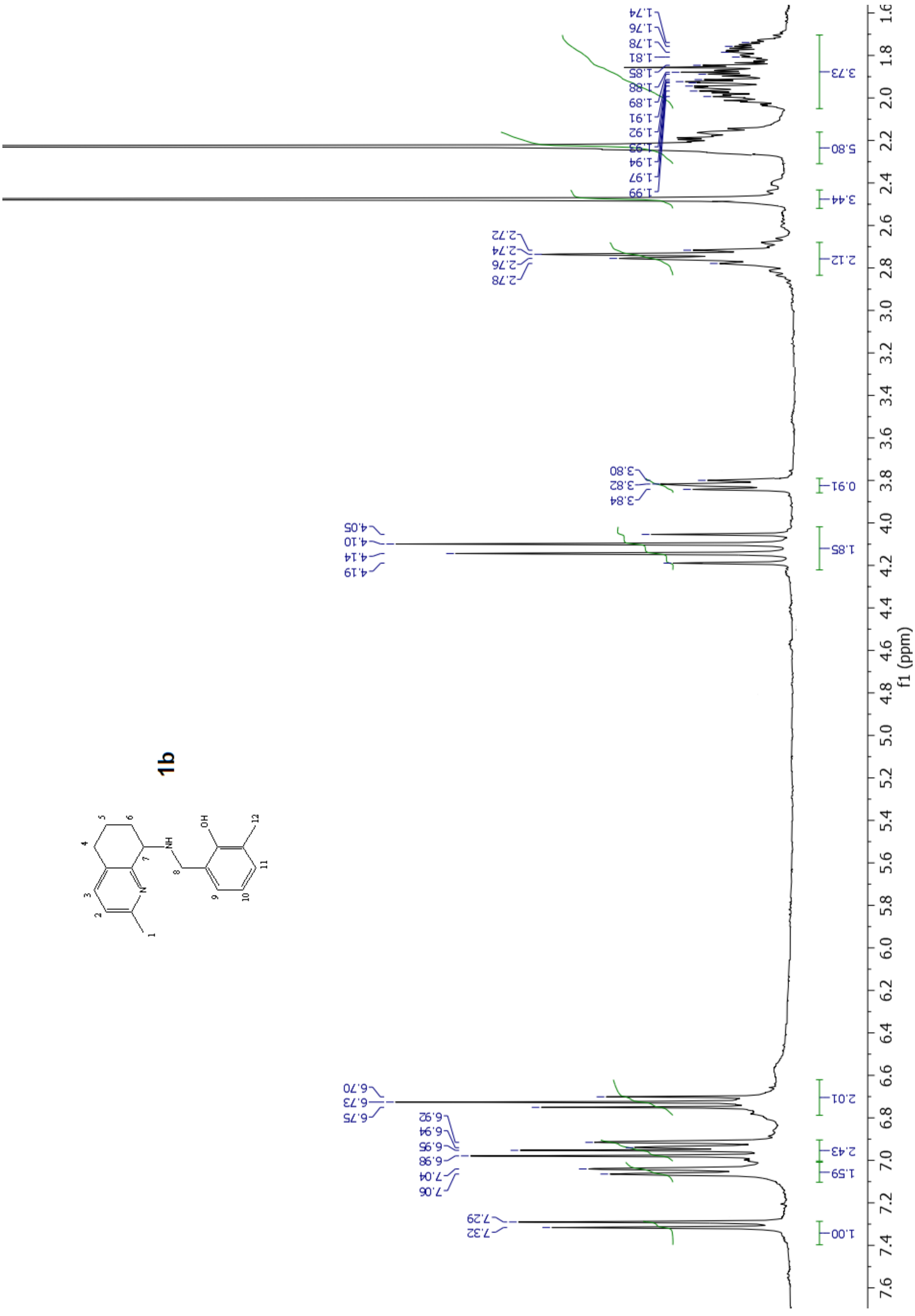

^{13}C-NMR in $CDCl_3$ (75 MHz) of 2-methyl-6-(((2-methyl-5,6,7,8-tetrahydroquinolin-8-yl)amino)methyl)phenol **1b**

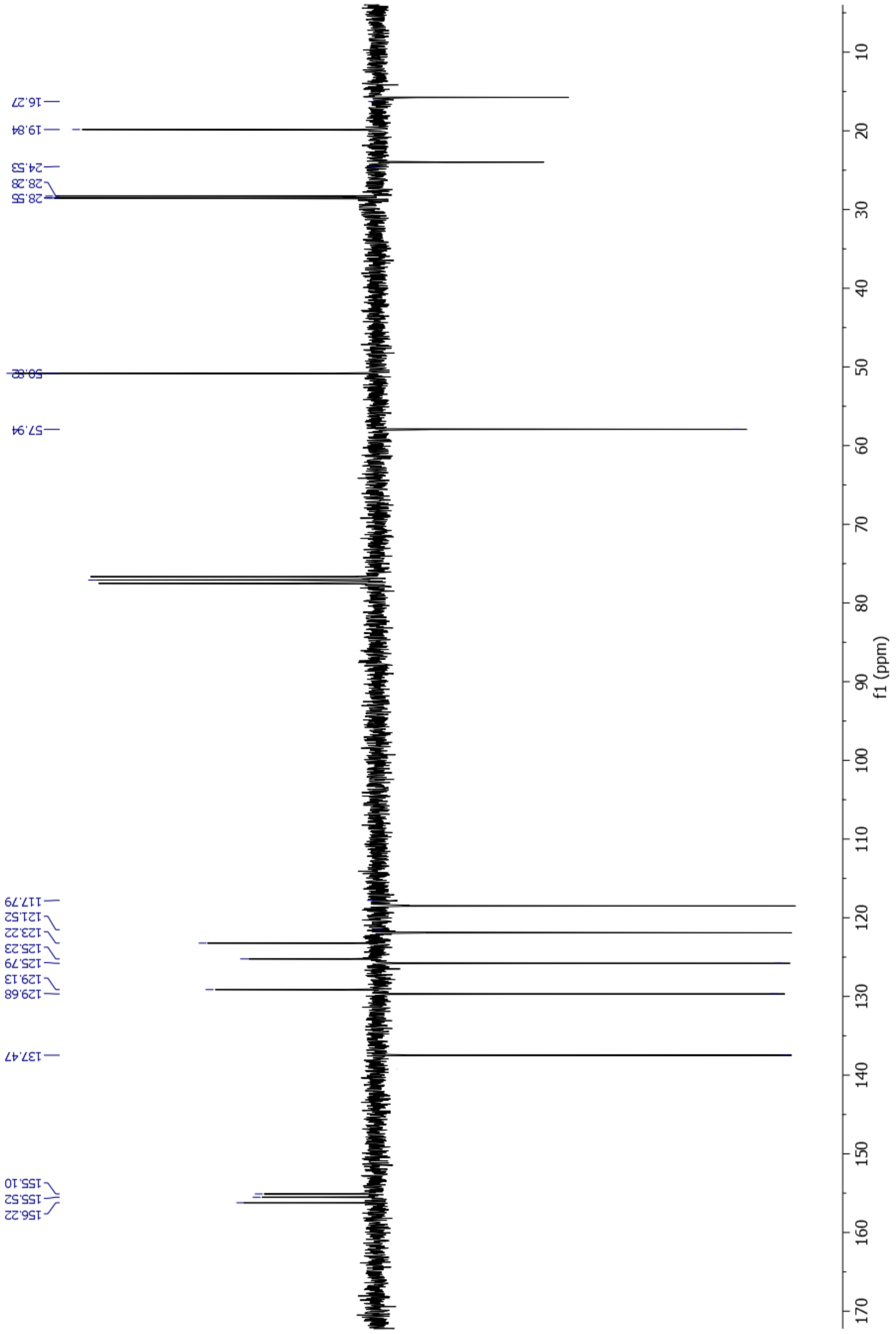

Log *P* of 2-methyl-6-(((2-methyl-5,6,7,8-tetrahydroquinolin-8-yl)amino)methyl)phenol **1b**

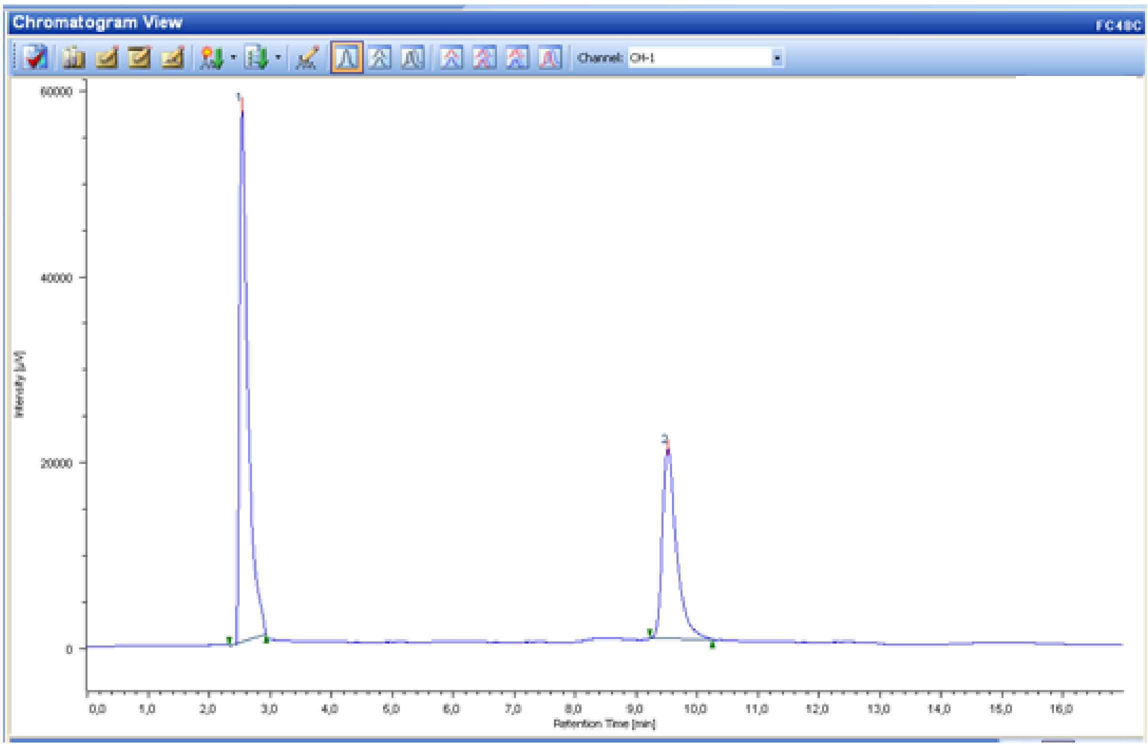

Peak Name	CH	tR	Area	Height	Area%	Height%	Quantity	NTP	Resolution	Symmetry Factor
Unknown	1	2,485	862136	21365	46,536	44,285	N/A	2458	3,125	2,025
Unknown	1	9,603	882436	10563	53,464	23,220	N/A	2156	N/A	1,985

Log $P_{o/w}$ = 3.96

^{1}H-NMR in $CDCl_3$ (300 MHz) of 4-methyl-2-(((2-methyl-5,6,7,8-tetrahydroquinolin-8-yl)amino)methyl)phenol **2b**

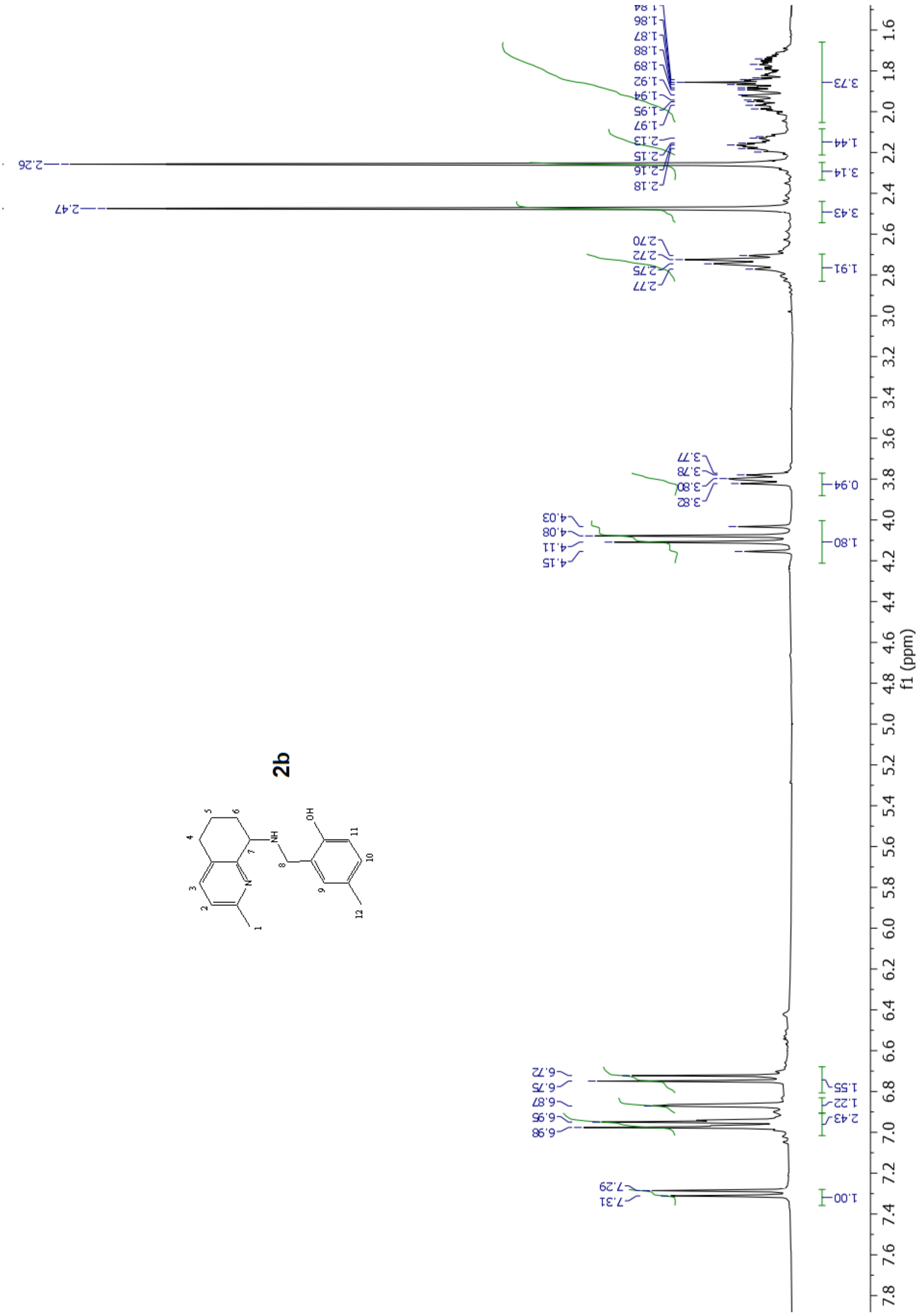

2b

^{13}C-NMR in $CDCl_3$ (75 MHz) of 4-methyl-2-(((2-methyl-5,6,7,8-tetrahydroquinolin-8-yl)amino)methyl)phenol **2b**

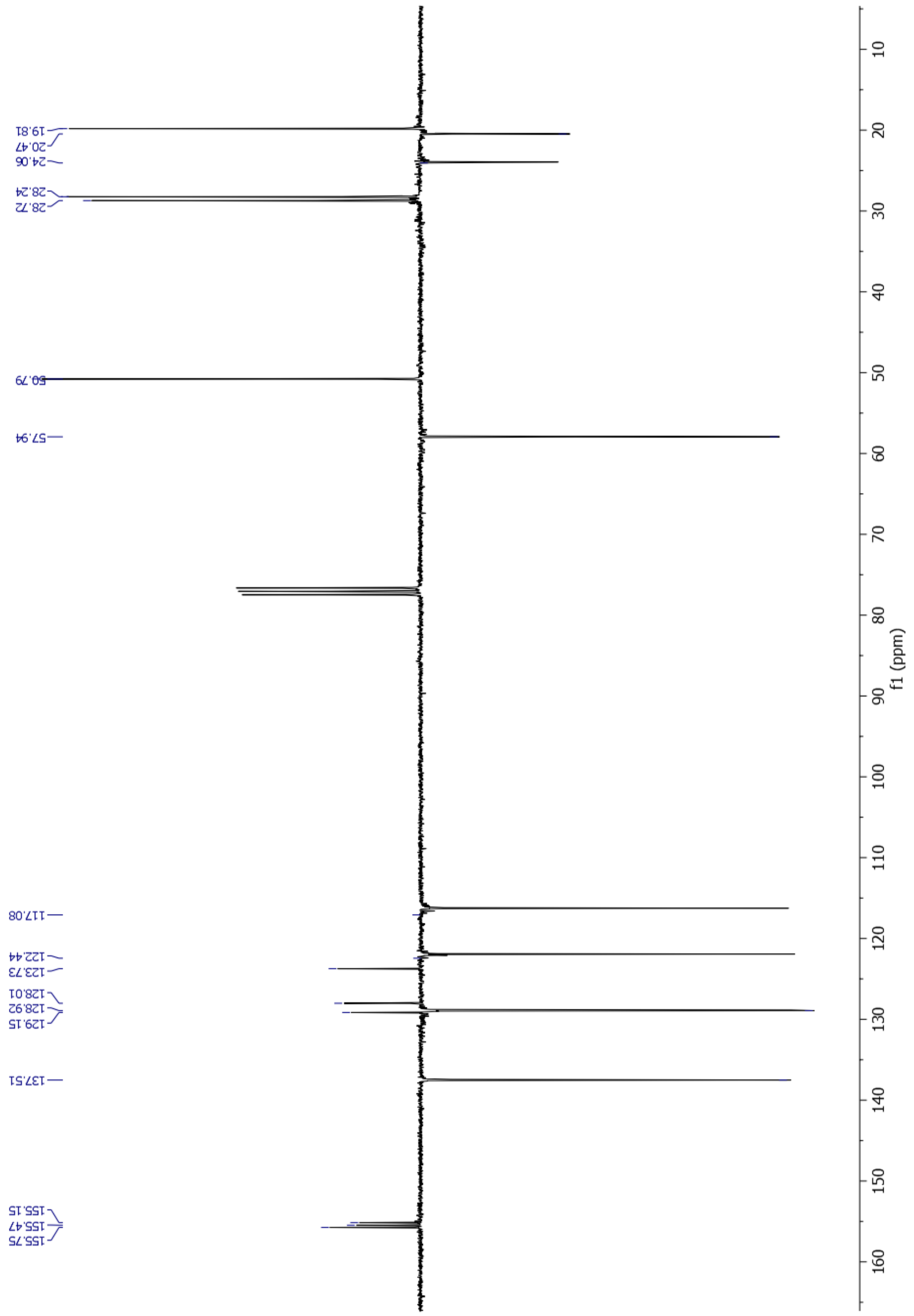

Log P of 4-methyl-2-(((2-methyl-5,6,7,8-tetrahydroquinolin-8-yl)amino)methyl)phenol **2b**

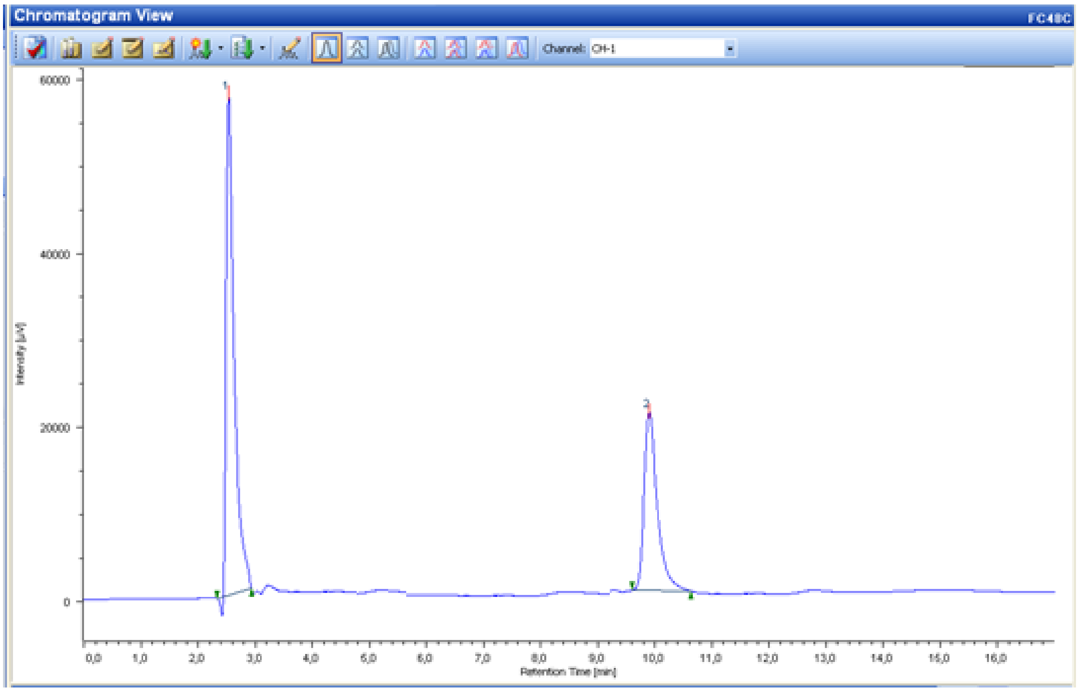

Peak Name	CH	tR	Area	Height	Area%	Height%	Quantity	NTP	Resolution	Symmetry Factor
Unknown	1	2,538	552946	67412	47,139	63,135	N/A	1763	6,716	2,207
Unknown	1	9,987	610006	13044	52,861	34,865	N/A	1608	N/A	2,151

Log $P_{o/w}$ = 4.03

^{1}H-NMR in $CDCl_3$ (300 MHz) of 2-methyl-N-(pyridin-2-ylmethyl)-5,6,7,8-tetrahydroquinolin-8-amine **3b**

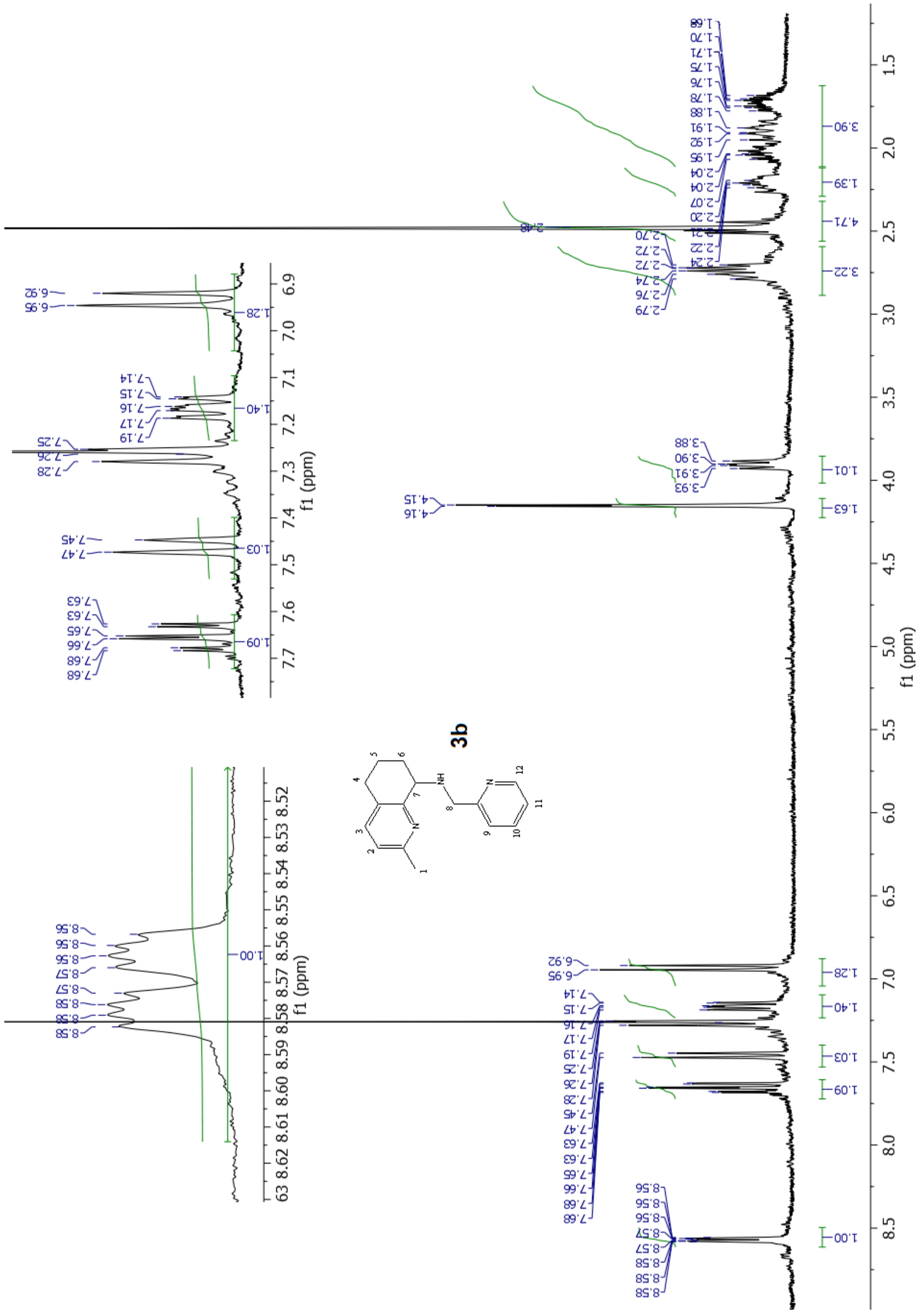

^{13}C-NMR in $CDCl_3$ (75 MHz) of 2-methyl-N-(pyridin-2-ylmethyl)-5,6,7,8-tetrahydroquinolin-8-amine
3b

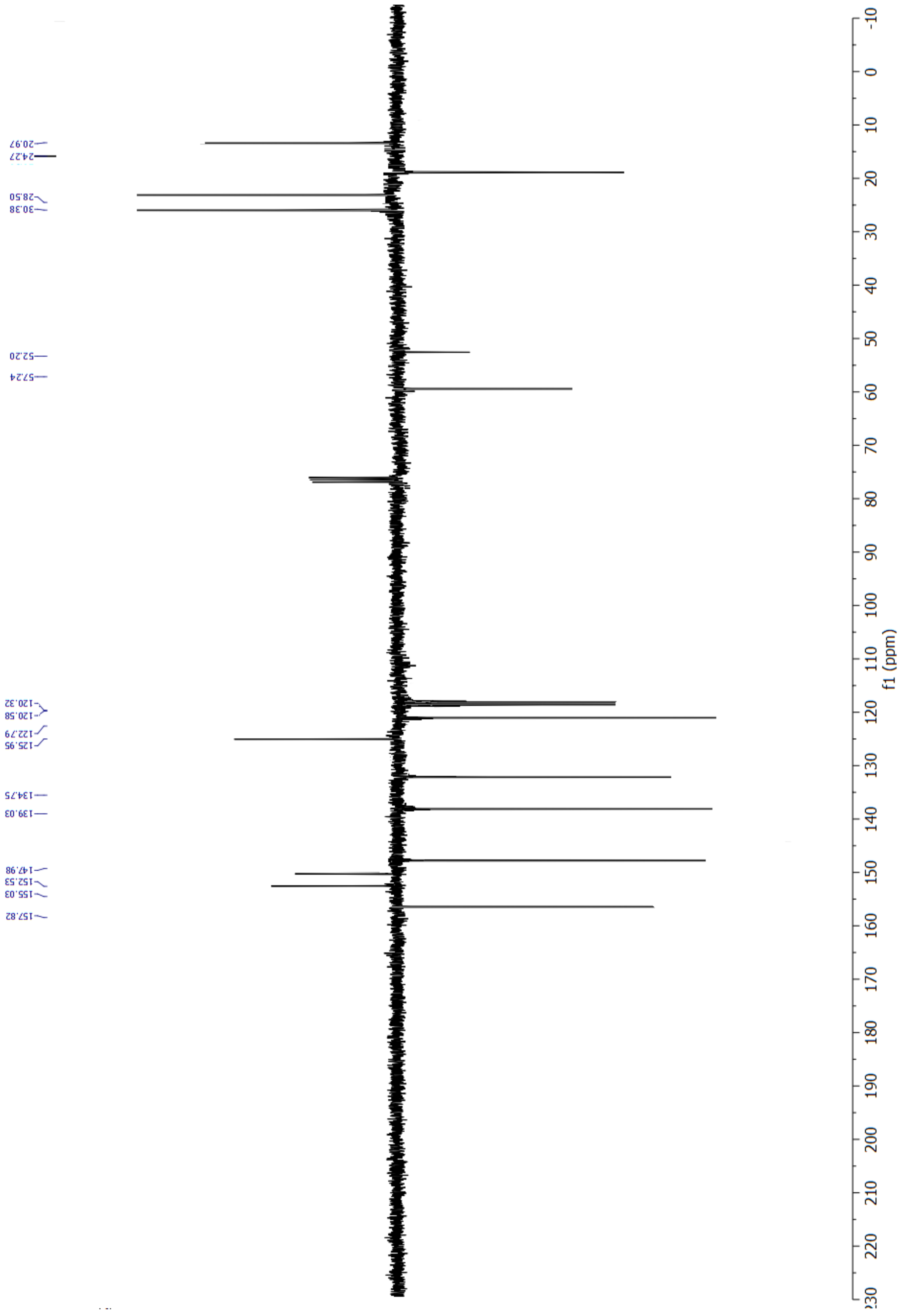

Log *P* of 2-methyl-N-(pyridin-2-ylmethyl)-5,6,7,8-tetrahydroquinolin-8-amine **3b**

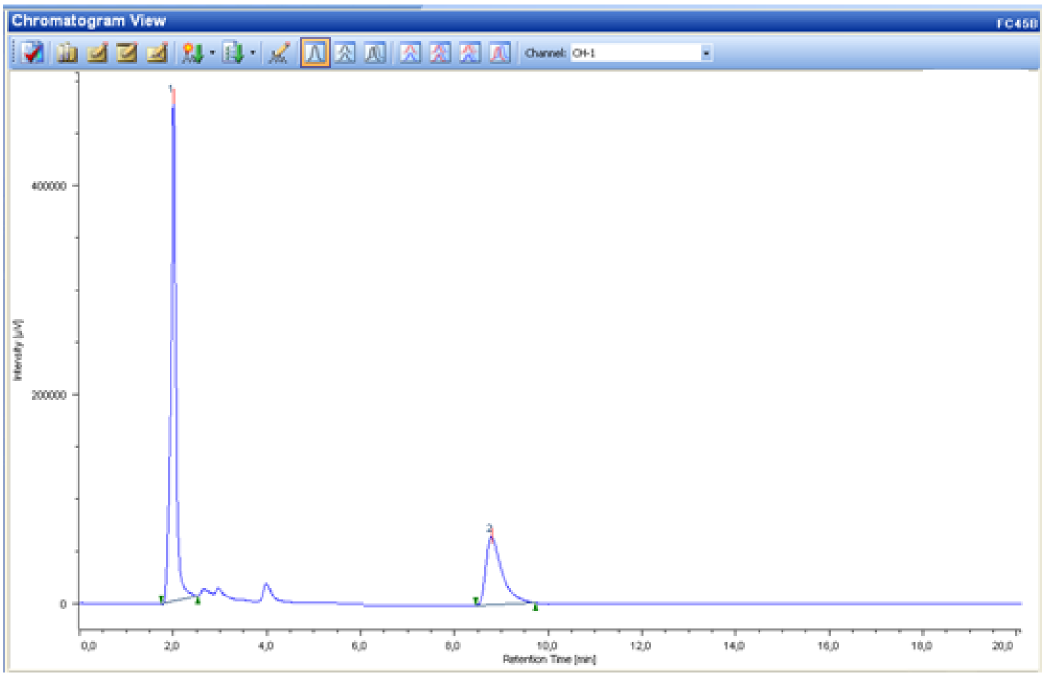

Peak Name	CH	tR	Area	Height	Area%	Height%	Quantity	NTP	Resolution	Symmetry Factor
Unknown	1	2,008	3675874	401608	69,885	86,100	N/A	2439	13,279	0,962
Unknown	1	8,967	1540967	66016	30,115	11,892	N/A	2291	N/A	1,004

Log $P_{o/w}$ = 4.12

^{1}H-NMR in $CDCl_3$ (300 MHz) of 2-methyl-N-((1-methyl-1H-imidazol-4-yl)methyl)-5,6,7,8-tetrahydroquinolin-8-amine **4b**

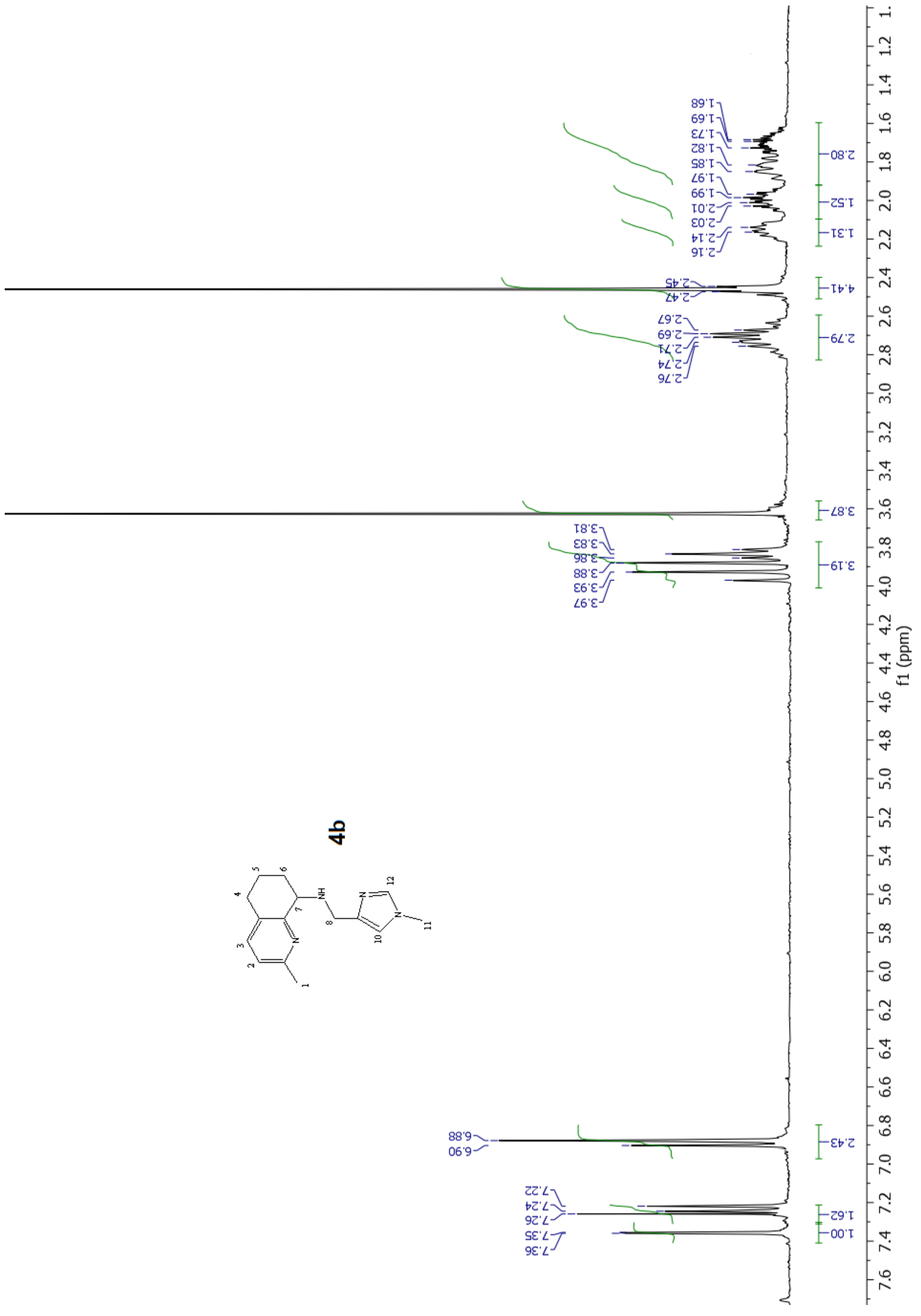

^{13}C-NMR in $CDCl_3$ (75 MHz) of 2-methyl-N-((1-methyl-1H-imidazol-4-yl)methyl)-5,6,7,8-tetrahydroquinolin-8-amine **4b**

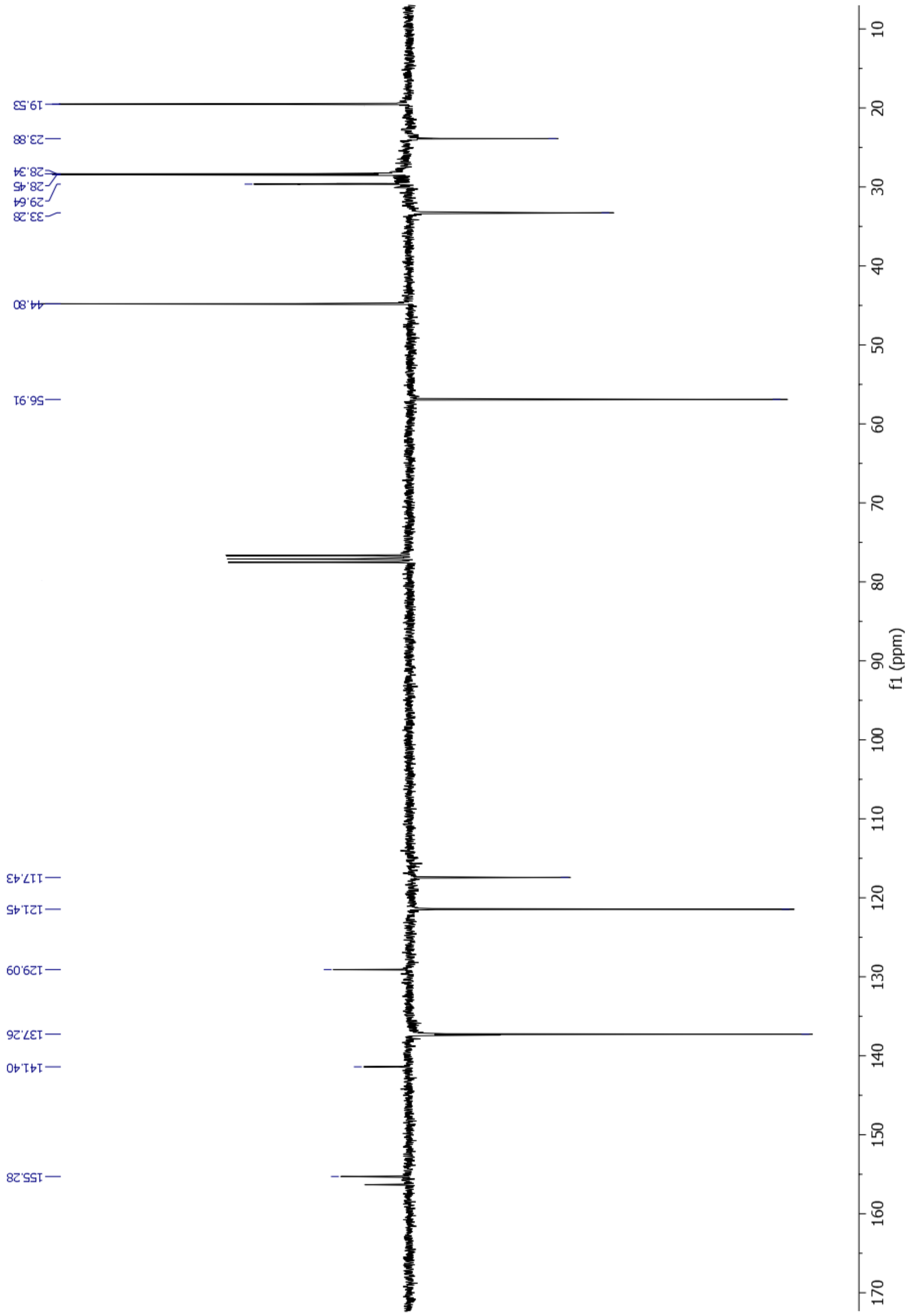

Log *P* of 2-methyl-N-((1-methyl-1H-imidazol-4-yl)methyl)-5,6,7,8-tetrahydroquinolin-8-amine **4b**

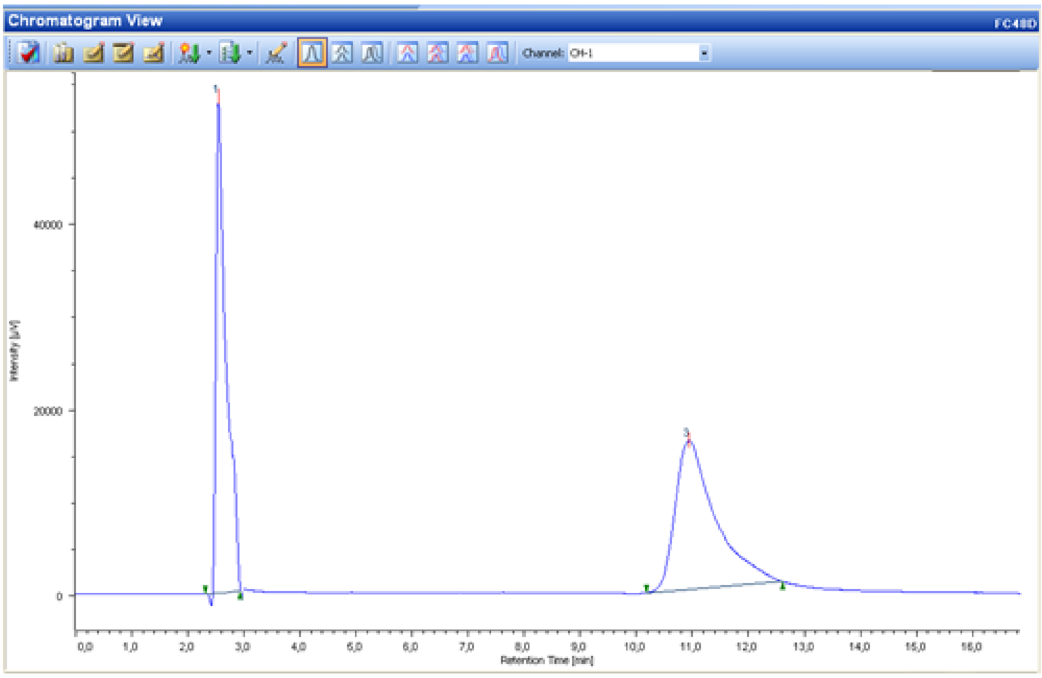

Peak Name	CH	tR	Area	Height	Area%	Height%	Quantity	NTP	Resolution	Symmetry Factor
Unknown	1	2,542	797608	63240	50,490	66,797	N/A	1160	4,443	3,516
Unknown	1	10,933	794327	15976	49,510	20,043	N/A	1135	N/A	1,871

Log $P_{o/w}$ = 4.11

^{1}H-NMR in $CDCl_3$ (300 MHz) of N-((1H-imidazol-2-yl)methyl)-2-methyl-5,6,7,8-tetrahydroquinolin-8-amine **5b**

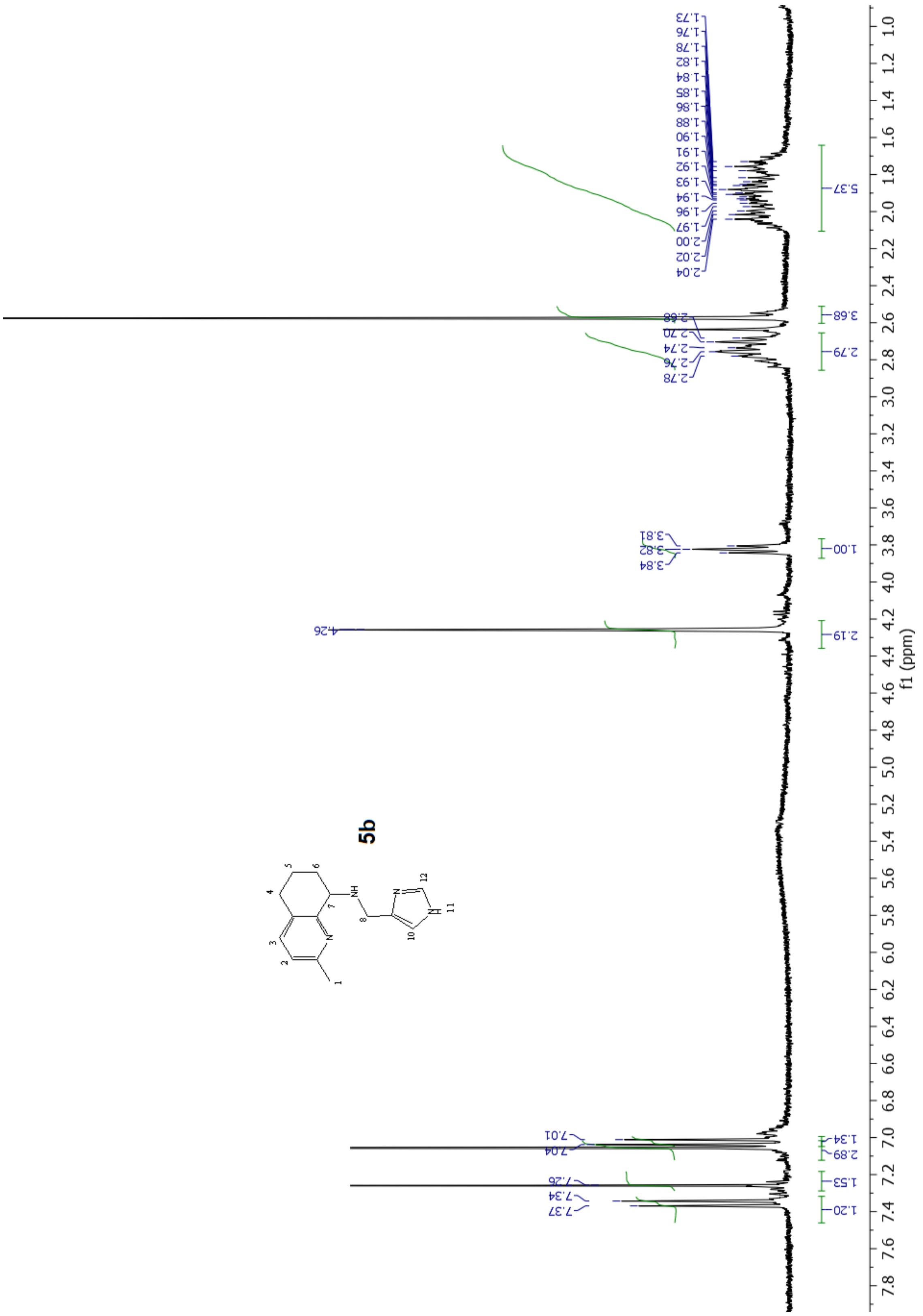

^{13}C-NMR in $CDCl_3$ (75 MHz) of N-((1H-imidazol-2-yl)methyl)-2-methyl-5,6,7,8-tetrahydroquinolin-8-amine **5b**

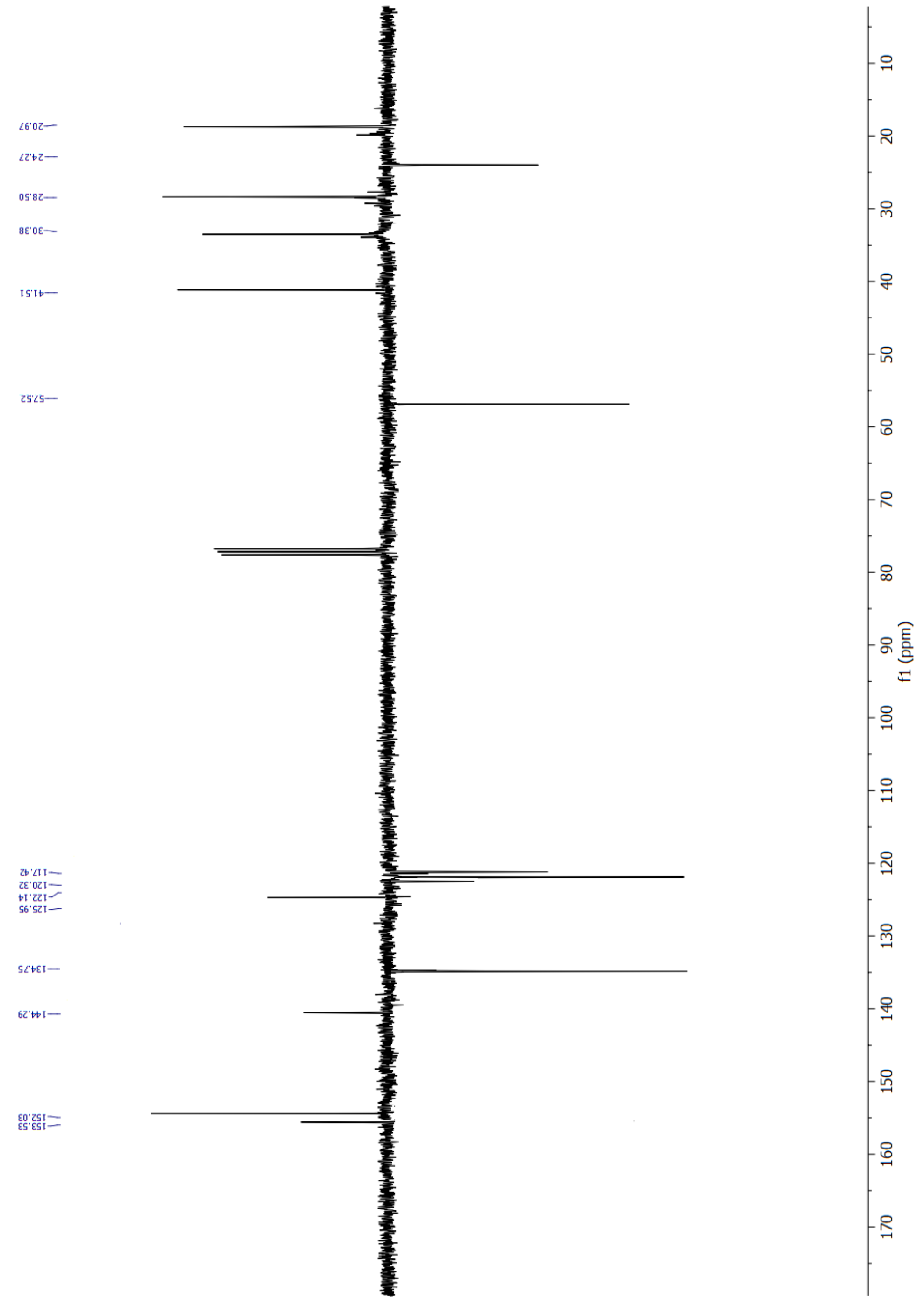

Log *P* of N-((1H-imidazol-2-yl)methyl)-2-methyl-5,6,7,8-tetrahydroquinolin-8-amine **5b**

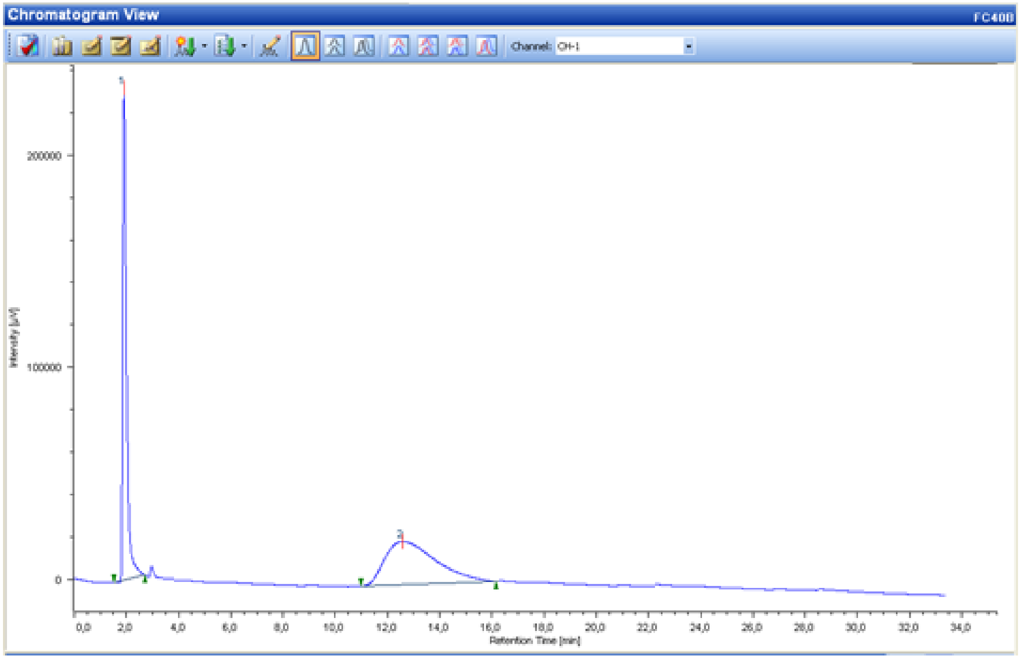

Peak Name	CH	tR	Area	Height	Area%	Height%	Quantity	NTP	Resolution	Symmetry Factor
Unknown	1	1,908	23986026	2312227	25,406	66,239	N/A	949	8,464	1,022
Unknown	1	12,358	49218765	16640	74,594	8,169	N/A	221	N/A	1,891

Log $P_{o/w}$ = 4.35

^{1}H-NMR in $CDCl_3$ (300 MHz) of 9-(((2-methyl-5,6,7,8-tetrahydroquinolin-8-yl)amino)methyl)-2,3,6,7-tetrahydro-1H,5H-pyrido[3,2,1-ij]quinolin-8-ol **6b**

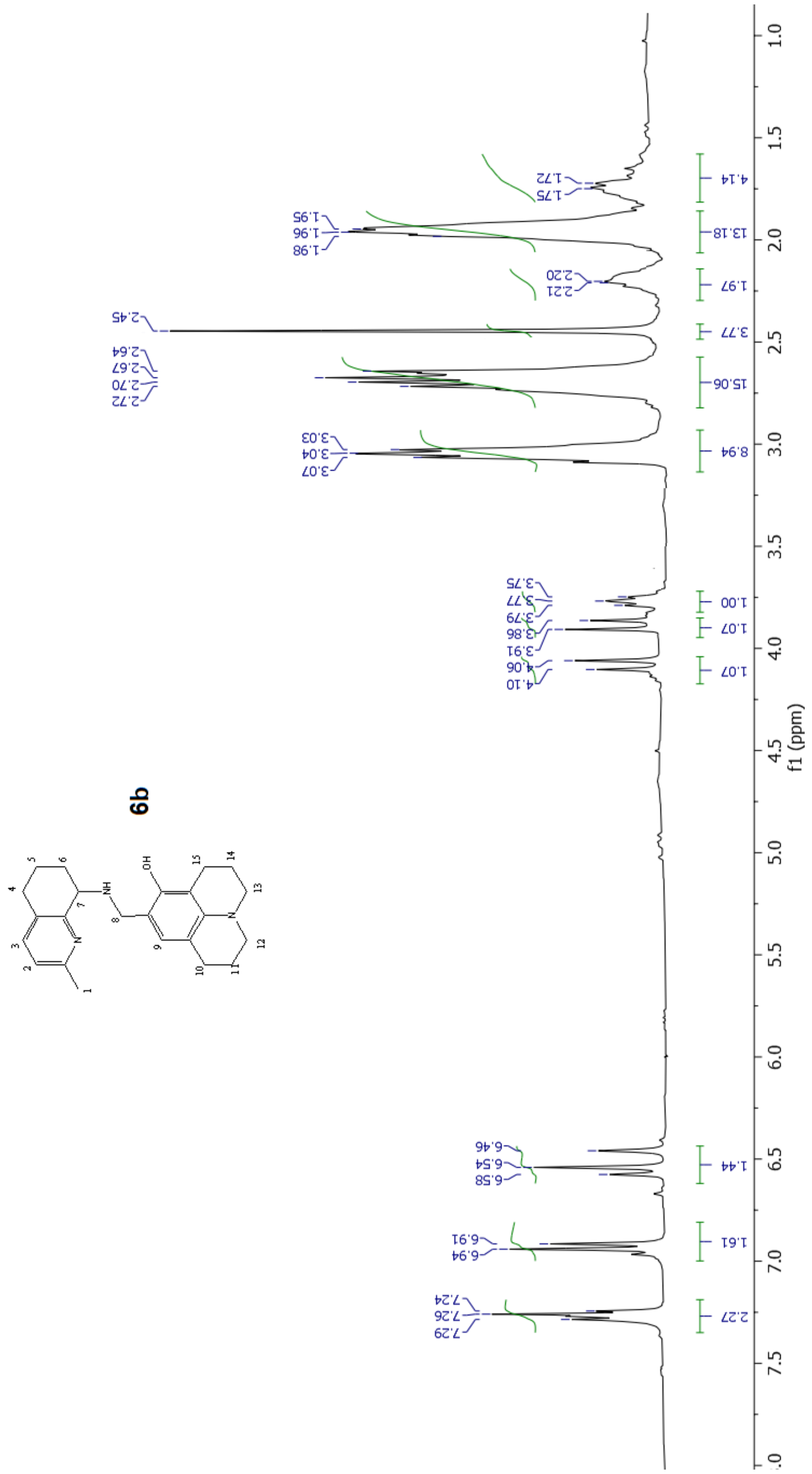

^{13}C-NMR in $CDCl_3$ (75 MHz) of 9-(((2-methyl-5,6,7,8-tetrahydroquinolin-8-yl)amino)methyl)-2,3,6,7-tetrahydro-1H,5H-pyrido[3,2,1-ij]quinolin-8-ol **6b**

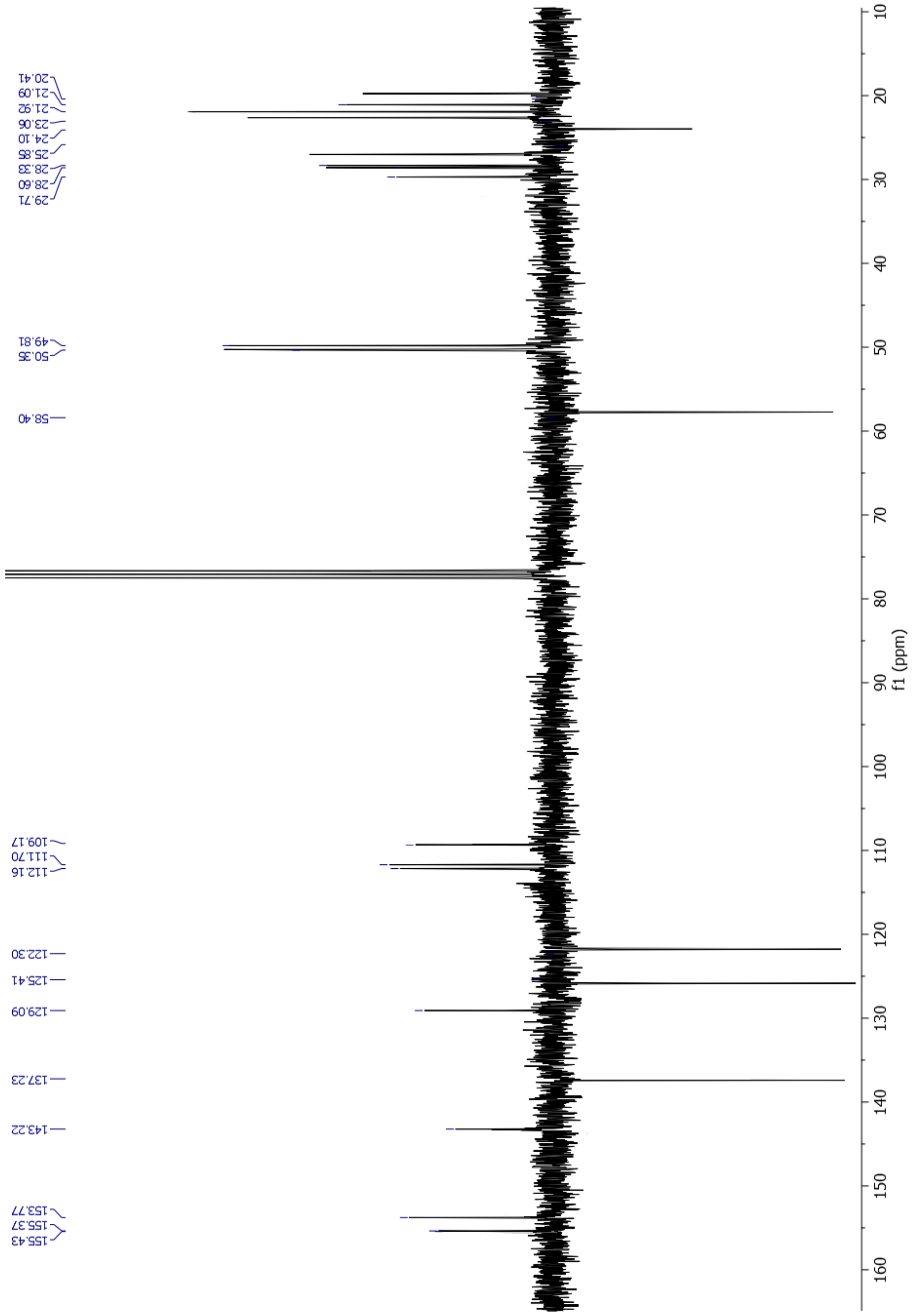

Log *P* of 9-(((2-methyl-5,6,7,8-tetrahydroquinolin-8-yl)amino)methyl)-2,3,6,7-tetrahydro-1H,5H-pyrido[3,2,1-ij]quinolin-8-ol **6b**

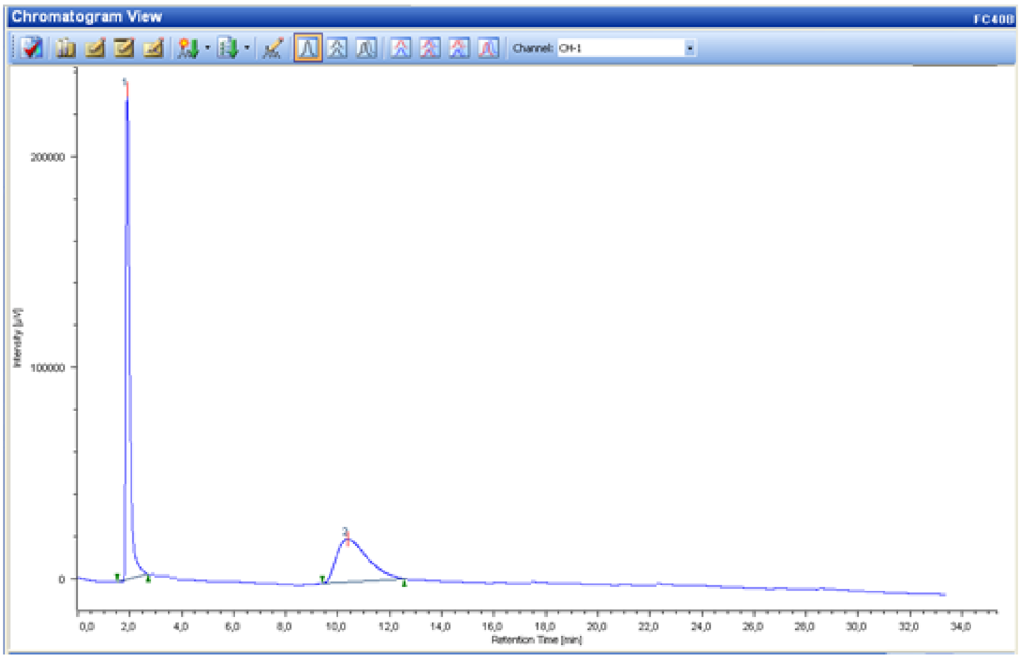

Peak Name	CH	tR	Area	Height	Area%	Height%	Quantity	NTP	Resolution	Symmetry Factor
Unknown	1	1,906	439682	23122	37,486	65,239	N/A	949	8,456	1,022
Unknown	1	10,467	573762	20266	62,514	27,692	N/A	423	1,130	1,601

Log $P_{o/w}$ = 4.13
